# Confirmation of high-throughput screening data and novel mechanistic insights into VDR-xenobiotic interactions by orthogonal assays

**DOI:** 10.1038/s41598-018-27055-3

**Published:** 2018-06-11

**Authors:** Debabrata Mahapatra, Jill A. Franzosa, Kyle Roell, Melaine Agnes Kuenemann, Keith A. Houck, David M. Reif, Denis Fourches, Seth W. Kullman

**Affiliations:** 1 Comparative Biomedical Sciences, College of Veterinary Medicine, North Carolina State University, Raleigh, North Carolina USA; 2 Department of Chemistry, Bioinformatics Research Center, North Carolina State University, Raleigh, North Carolina USA; 3 Department of Biological Sciences, North Carolina State University, Raleigh, North Carolina USA; 4 Program in Environmental and Molecular Toxicology, North Carolina State University, Raleigh, North Carolina USA; 5 National Center for Computational Toxicology, Office of Research and Development, U.S. Environmental Protection Agency, RTP, Raleigh, North Carolina USA

## Abstract

High throughput screening (HTS) programs have demonstrated that the Vitamin D receptor (VDR) is activated and/or antagonized by a wide range of structurally diverse chemicals. In this study, we examined the Tox21 qHTS data set generated against VDR for reproducibility and concordance and elucidated functional insights into VDR-xenobiotic interactions. Twenty-one potential VDR agonists and 19 VDR antagonists were identified from a subset of >400 compounds with putative VDR activity and examined for VDR functionality utilizing select orthogonal assays. Transient transactivation assay (TT) using a human VDR plasmid and Cyp24 luciferase reporter construct revealed 20/21 active VDR agonists and 18/19 active VDR antagonists. Mammalian-2-hybrid assay (M2H) was then used to evaluate VDR interactions with co-activators and co-regulators. With the exception of a select few compounds, VDR agonists exhibited significant recruitment of co-regulators and co-activators whereas antagonists exhibited considerable attenuation of recruitment by VDR. A unique set of compounds exhibiting synergistic activity in antagonist mode and no activity in agonist mode was identified. Cheminformatics modeling of VDR-ligand interactions were conducted and revealed selective ligand VDR interaction. Overall, data emphasizes the molecular complexity of ligand-mediated interactions with VDR and suggest that VDR transactivation may be a target site of action for diverse xenobiotics.

## Introduction

Following National Research Council’s recommendations^1^ for a shift from traditional low throughput *in vivo* rodent assays to less expensive *in vitro* high throughput methods, core regulatory bodies such as the U.S. Environmental Protection Agency (EPA), National Toxicology Program (NTP), National Institute of Health (NIH), NIH National Center for Advancing Translational Sciences (NCATS), US Food and Drug Administration (FDA) responded to the urgency with the initiation of ToxCast^TM^ and Tox21 programs^2,3^. These programs were aimed at prioritizing toxicity evaluations through promoting the increasing use of *in vitro* high throughput screening assays for large numbers of chemicals already in commercial use for which little or no toxicity data was available^4,5^. These initiatives have now resulted in the generation of an enormous, publicly available compendium of chemical-biological interactions that has enabled researchers to infer predictive public health decisions.

Within both the ToxCast^TM^ and Tox21 programs, disruption in nuclear receptor (NR) signaling represents a defined set of molecular targets of interest. Given the role of NR’s in modulating specific endocrine functions, assessing chemical interactions with this superfamily of proteins provides mechanistic data that enables predictive assessments of toxicity pathways related to human disease. Subsequently, targeted cell based *in vitro* studies have been conducted to identify the selectivity, potency and efficacy of environmentally relevant chemicals that can modify receptor function. For instance, assessments of estrogen receptor alpha (ERα) agonists/antagonists demonstrated the feasibility of quantitative high throughput assays to identify environmental chemicals with the potential to interact with ERα and revealed the importance of both known and novel ERα active structure classes as agonists/antagonists^6^. Similarly, structure-activity relationships of FXR-active compounds suggest that this receptor may have multiple modes of action that modulate receptor-coregulator interactions essential to NR transactivation^7^. Recent studies have also utilized computational modeling based approaches to build predictive models based on structural information and activity data^8^. Consistent within these approaches is the observation that receptor-ligand molecular interactions are mediated through specific structural determinants that modulate receptor conformation and thus transactivational capacity.

In the wake of the above-mentioned targeted NR studies, and the emergence of newly identified environmental compounds with potential endocrine disrupting properties, we focused our attention to the library of screened compounds that altered the transactivational activity of vitamin D receptor (VDR). Vitamin D has gained much attention in recent years not only for its role in classical bone and mineral homeostatic functions but also for its roles in neurodevelopment, neuroprotection, cell proliferation and differentiation, immune function and inflammation. Vitamin D is unique in that in its native state it is a vitamin or an essential dietary component. However, upon metabolic activation it is converted to 1α,25-dihydroxyvitamin D_3_ (1,25D_3_, calcitriol) and serves a well-defined endocrine function as a steroid hormone^9^. Classical transcriptional actions of 1,25D_3_ are mediated through its high affinity interactions with the vitamin D receptor (VDR). VDR is a member of the nuclear receptor superfamily, which is comprised of a large group of ligand-activated transcription factors. The mechanism of VDR-mediated gene transcription closely resembles that of other steroid hormones usually involving high affinity interactions between ligand and receptor, heterodimerization with RXR, association with a canonical vitamin D response element (VDRE) within target promoter regions and recruitment of co-regulatory proteins, members of the MED complex and RNA polymerase II to initiate both transactivation and transrepression of gene regulatory networks critical to cellular processes^10^. Similar to other steroid hormones, 1,25D_3_ has a short half-life and optimum blood levels are maintained by a tight feedback mechanism through the action of catabolic enzyme CYP24A1. 1,25D_3_ also serves paracrine/autocrine functions since several target tissues^11^ are capable of synthesizing the active form of the hormone^12^. Accordingly, deficiency of vitamin D affects a variety of organs and systems resulting in growth retardation and skeletal deformities, and increased risk of chronic diseases including common cancers, autoimmune, infectious and cardiovascular diseases and neuropsychiatric disorders^9,13^.

In this study, we examined the Tox21 qHTS data set generated against VDR (see materials and methods) for reproducibility and concordance in a low throughput format and investigated VDR receptor activity profiles *in vitro* using luciferase reporter gene assays. We examined how structurally and functionally diverse compounds included in the Tox21 chemical space modify core nuclear receptor functions of VDR with respect to VDR heterodimerization with human RXRα, recruitment of coactivator (SRC-1) or corepressor (NCoR), and the ability to initiate/inhibit transactivation of CYP24A1. Molecular modeling was also employed to forecast and study the molecular interactions of the most potent compounds once docked in the VDR binding site.

## Results

### Selection of putative VDR agonists and antagonists for orthogonal screening

**Figure 1:**
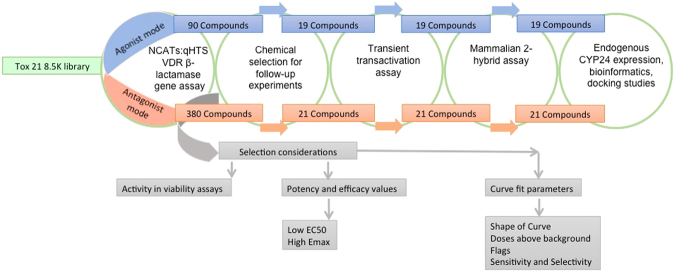
Schematic overview of compound selection criteria and experimental workflow.

Experimental qHTS screening results of Tox21 library compounds in VDR β-lactamase reporter agonist and antagonist assays were used to prioritize and select compounds for screening in our orthogonal *in vitro* assays^14^. Results from the Tox21 curve-fitting analysis suggest that the human VDR is activated and/or antagonized by a wide range of structurally diverse chemicals including naturally occurring, synthetic, and environmental chemicals. In agonist-mode, over 90 compounds activated the VDR reporter gene assay. Over 380 potential VDR antagonists were also identified with (AC_50_) values ranging from sub-micromolar to 50 µM. To select the subset of compounds for further *in vitro* testing, the AC_50_ values from the VDR beta-lactamase reporter assay and the cell viability assay were used to calculate a ratio (AC_50viability_/AC_50VDR_). The list of active agonists and antagonists was reduced to compounds with ratio values greater than 5 and assessed in relation to curve fit parameters and flag criteria, which are available via the ToxCast Dashboard (https://actor.epa.gov/dashboard/). In total, 21 agonists and 19 antagonists were selected for further screening in orthogonal VDR screening assays (Fig. 1).

### Ligand-induced receptor transactivation

**Figure 2:**
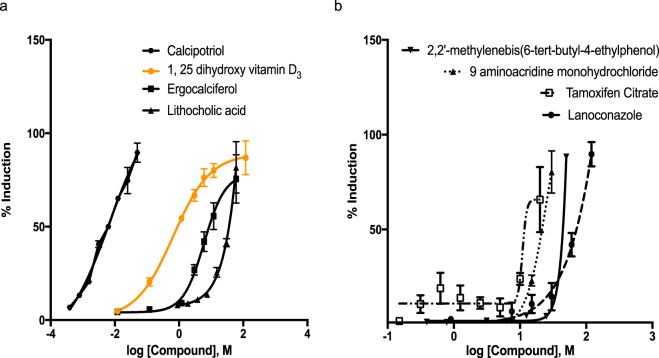
Representative concentration response curves of select VDR agonists identified by transient transactivation assay (CYP24A1-Luc). Concentration response curves of (**a**) Vitamin D_3_ and related active analogs: Calcipotriol (AC_**50**_ = 0.0009 µM), 1–25 dihydroxy vitamin D_3_ (AC_**50**_ = 0.65 nM), Ergocalciferol (EC_**50**_ = 14.44 µM), LCA (EC_**50**_ = 16.82 µM); (**b**) Less active agonists: 2,2′-methylenebis(6-tert-butyl-4-ethylphenol) (EC_**50**_ = 36.76 µM), 9 aminoacridine monohydrochloride (EC_**50**_ = 12.58 µM), Tamoxifen Citrate (EC_**50**_ = 3.84 µM), Lanoconazole (EC_**50**_ = 20.99 µM). Assays were run in HEK293T cells and data expressed as mean ± SEM (n = 3).

Selected compounds, 21 agonists and 19 antagonists, were screened for functional nuclear receptor activities using human VDR expression constructs. Our approach to validate target receptor interaction incorporated fundamental components of nuclear receptor function including VDR transactivation and co-regulator recruitment. Each of these critical components of NR function were essential in demonstrating that receptor “agonists” or receptor “antagonists” facilitate molecular interactions necessary for target gene induction or gene repression *in vivo*. Transient transactivation assays in HEK293T cells were utilized to assess selected compounds in either agonist or antagonist modes. AC_50_ values for VDR agonists in this study were determined relative to 1,25D_3_ as a positive control. From a total of 21 selected Tox21 agonists, 20 were confirmed to exhibit VDR transactivation activities. Figure 2 illustrates concentration responses for select VDR agonists with Table 1 reporting all AC_50_ and E_max_ values (also see supplemental data from remaining concentration response modeling results). Overall, compounds exhibited a wide spectrum of activity as evident by their derived AC_50_ values that ranged from 0.009 μM for calcipotriol up to 37.41 μM for novaluron. One compound, falnidamol hydrochloride consistently failed to exhibit any concentration response interactions resulting in an ambiguous and inconsistent AC_50_ value after several repeated trials. This compound however was found to be active in the Tox21 qHTS data set.

**Table 1:** Tested compounds with their names, Cas numbers, origin, activity, AC_50_ values obtained from Tox21 qHTS data set and orthogonal (Transient transactivation assay) efficacy values, with their corresponding concentration ranges.

Compound Name/CAS # Agonists	Origin	Activity	Tox21 AC_50_	Derived AC_50_ (Transient transactivation assay)	Derived Efficacy (fold induction)	Concentration range (µM)
7-(Dimethylamino)-4-methylcoumarin (91–44–1)	EPA	Active	2.88–21.5*	0.7934	17.3255	0.01–15 µM
Disodium 4,4′-bis(2-sulfostyryl) biphenyl (27344-41-8)	EPA	Active	18	0.3935	9.4578	0.09–120 µM
4-Aminofolic acid (Aminopterin) (54-62-6)	EPA	Active	11.8	33.7346	16.3718	0.09–120 µM
Ergocalciferol (50-14-6)	EPA	Active	4.28–9.02*	14.4457	82.6111	0.09–60 µM
Alpha-Terthiophene (1081-34-1)	EPA	Active	15.7	0.3048	19.9132	0.09–120 µM
Triamterene (396-01-0)	EPA	Active	5.3–42.1*	3.0672	14.7020	0.09–40 µM
Novaluron (116714-46-6)	EPA	Active	23.4	37.4130	16.3718	0.09–120 µM
2,2′-methylenebis(6-tert-butyl-4-ethylphenol) (88-24-4)	SIGMA	Active	52.6	36.7667	89.3748	0.09–120 µM
9 Aminoacridine monohydrochloride (52417-22-8)	SIGMA	Active	4.25–11.1*	12.5863	44.5165	0.09–30 µM
2,2′-methylenebis(6-tert-butyl-4-methylphenol) (119-47-1)	SIGMA	Active	32.1	9.3229	29.470	0.09–40 µM
4,4′-butylidenebis(6-tert-butyl-m-cresol (96-69-5)	SIGMA	Active	20.6	0.293734005	13.4171	0.09–20 µM
Tamoxifen citrate (54965-24-1)	SIGMA	Active	33–56*	3.8490	20.5748	0.09–20 µM
Methyl 3-amino-5,6-dichloropyrazine-2-carboxylate (1458-18-0)	SIGMA	Active	8.78	0.4457	44.5165	0.09–1.5 µM
2,7 Naphthalene disulfonic acid (312693-54-2)	EPA	Active	14.5	13.7882	14.1669	0.09–120 µM
Cridanimod (38609-97-1)	EPA	Active	11.1	13.8855	19.1624	0.09–120 µM
7 methyl benzo (a) pyrene (63041-77-0)	SIGMA	Active	2.54	10.6287	19.1624	0.09–120 µM
Benzenesulfonic acid (98-11-3)	SIGMA	Active	9.9	0.7719	13.6777	0.09–120 µM
Falnidamol dihydrochloride (1216920-18-1)	SIGMA	Inactive	3.3	NA	9.9267	0.09–0.9 µM
Lithocholic acid (434-13-9)	SIGMA	Active	5.88–6.69*	22.2952	44.51651	0.09–50 µM
Calcipotriol (112965-21-6)	SIGMA	Active	0.000294	0.00997	101.7383	0.39nM-0.05 µM
Lanoconazole (101530-10-3)	SIGMA	Active	21.1	20.9946	29.4706	0.09–120 µM
**Compound Name/CAS #** **Antagonists**	**Origin**	**Activity**	**Tox21 IC**_**50**_	**Derived IC**_**50**_ **(Transient transactivation assay)**	**Derived Efficacy (fold inhibition)**	**Concentration range (**µ**M)**
Dibutyltin dichloride (683-18-1)	EPA	Active	0.0823	0.5422	20.5748	0–1 µM
Triphenyltin hydroxide (76-87-9)	EPA	Active	0.0929	0.0454	89.3748	0–1 µM
Ziram (137-30-4)	EPA	Active	1.39	1.0011	89.3748	0–6 µM
Fluorescein sodium (518-47-8)	EPA	Inactive	0.356	NA	101.7383	0–12 µM
Cadmium chloride (10108-64-2)	EPA	Active	0.171	0.3797	44.5165	0–12 µM
Potassium dicyanoaurate (13967-50-5)	EPA	Active	0.0912	0.0985	76.1807	0–6 µM
Cadmium dinitrate (10325-94-7)	EPA	Active	0.167	1.3384	82.6111	0–12 µM
Tributyltin chloride (1461-22-9)	SIGMA	Active	0.9594	0.2005	89.3748	0–1 µM
Thiram (137-26-8)	SIGMA	Active	1.31–0.3	0.4284	76.1807	0–12 µM
Aristolochic acid (10190-99-5)	EPA	Active	7.32	0.8267	76.1807	0–12 µM
Proflavine hydrochloride (952-23-8)	EPA	Active	3.41	0.5554	68.3825	0–12 µM
Tazobactam sodium (89785-84-2)	EPA	Active	1.98	0.2833	79.1108	0–12 µM
Carfizomib (868540-17-4)	EPA	Active	0.7481	0.5377	80.6485	0–12 µM
Phenylarsine oxide (637-03-6)	SIGMA	Active	0.097498	0.0128	89.3748	0–0.37 µM
Proscillaridin (466-06-8)	SIGMA	Active	0.04335	1.8974	14.1669	0–12 µM
Chlorambucil (305-03-3)	SIGMA	Active	0.000058	0.0210	68.5251	0–0.37 µM
Cadmium acetate dihydrate (5743044)	SIGMA	Active	0.271	1.1667	20.5478	0–12 µM
Cadmium reference solution (7440-43-9)	SIGMA	Active	NA	0.1984	76.1807	0–2.75 nM
Dichlone (117-80-6)	SIGMA	Active	0.512–0.418	0.2428	86.9798	0–1.5 µM
Menadiol (481-85-6)	SIGMA	Active	0.979	3.4960	101.7383	0–12 µM

Note that some of the Tox21 qHTS AC_50_ values have a range (*). The efficacy values were derived from the top asymptote of the corresponding Hill or Gain-Loss model. They represent the maximum response for a given agonist or antagonist (fold induction or fold inhibition respectively).

**Figure 3:**
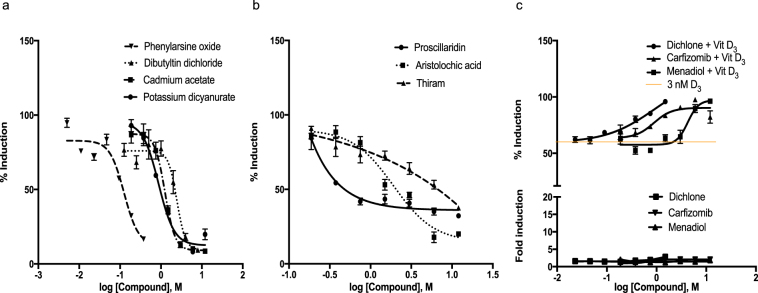
Representative concentration response curves of select VDR antagonists identified by transient transactivation assay (Cyp24-Luc). Concentration response curves of (**a**) Metal containing compounds: Phenylarsine oxide (IC_**50**_ = 0.012 µM), Dibutyltin (IC_**50**_ = 0.54 µM), Potassium dicyanurate (IC_**50**_ = 0.09 µM), Cadmium acetate (IC_**50**_ = 1.16 µM); (**b**) Non-metal containing compounds: Proscillaridin (IC_**50**_ = 1.89 µM), Aristocholic acid (IC_**50**_ = 0.82 µM), Thiram (IC_**50**_ = 0.42 µM), (**c**) Reverse agonists Carfizomib (IC_**50**_ = 0.53 µM), Dichlone (IC_**50**_ = 0.24 µM), and Menadiol (IC_**50**_ = 3.49 µM). Assays were run in HEK293T cells in the presence of 3 nM 1,25D_3_ and data expressed as Mean ± SEM (n = 3).

The activity of VDR antagonists was assessed through quantifying the inhibition of 1,25D_3_-induced (3 nM) responses in transient transactivation assays. Eighteen out of a total of 19 compounds were found to be functionally active with the exception of fluorescein sodium. Activity of compounds ranged widely with AC_50_ values ranging between 0.01μM for phenylarsine oxide up to 7.32 μM for aristolochic acid. Compounds that contained a transition metal atom (*i.e*., Cd, Tin, Au, Ar) exhibited both greater potency and efficacy on the VDR activity than those without metals (Fig. 3a). The trialkyltins, ziram (zinc containing pesticide) and other cadmium salts followed a similar trend. Non-metal containing compounds (i.e., thiram, aristolochic acid and proscillaridin) were relatively less potent and efficacious antagonists (Fig. 3b). A unique group of three non-metal containing compounds, namely dichlone, carfizomib and menadiol were identified that exhibited very weak agonist activity when tested alone but exhibited moderate to marked synergistic activity when tested in antagonist mode in the presence of 1,25D_3_ (Fig. 3c). These compounds were categorized as antagonists in the Tox21 qHTS database.

### Cheminformatics modeling of VDR-ligand interactions

Three-dimensional molecular docking studies were conducted for each active compound identified in our screening assays, so that we could evaluate and better understand their respective binding modes in the VDR active site. Since the first co-crystalized structure of the VDR receptor with 1,25D_3_ was reported in 2000 by Rochel *et al*.^15^, multiple X-ray structures of the VDR receptor in complex with different small molecule ligands^16–19^ have been published and deposited in the online Protein Data Bank. The thorough analysis of the different holo crystal structures did not reveal any significant conformational and/or structural changes^20^. As a result, we decided to select the recent X-ray structure for the human VDR co-crystalized with calcipotriol^19^ (PDB code: 1S19).

**Figure 4:**
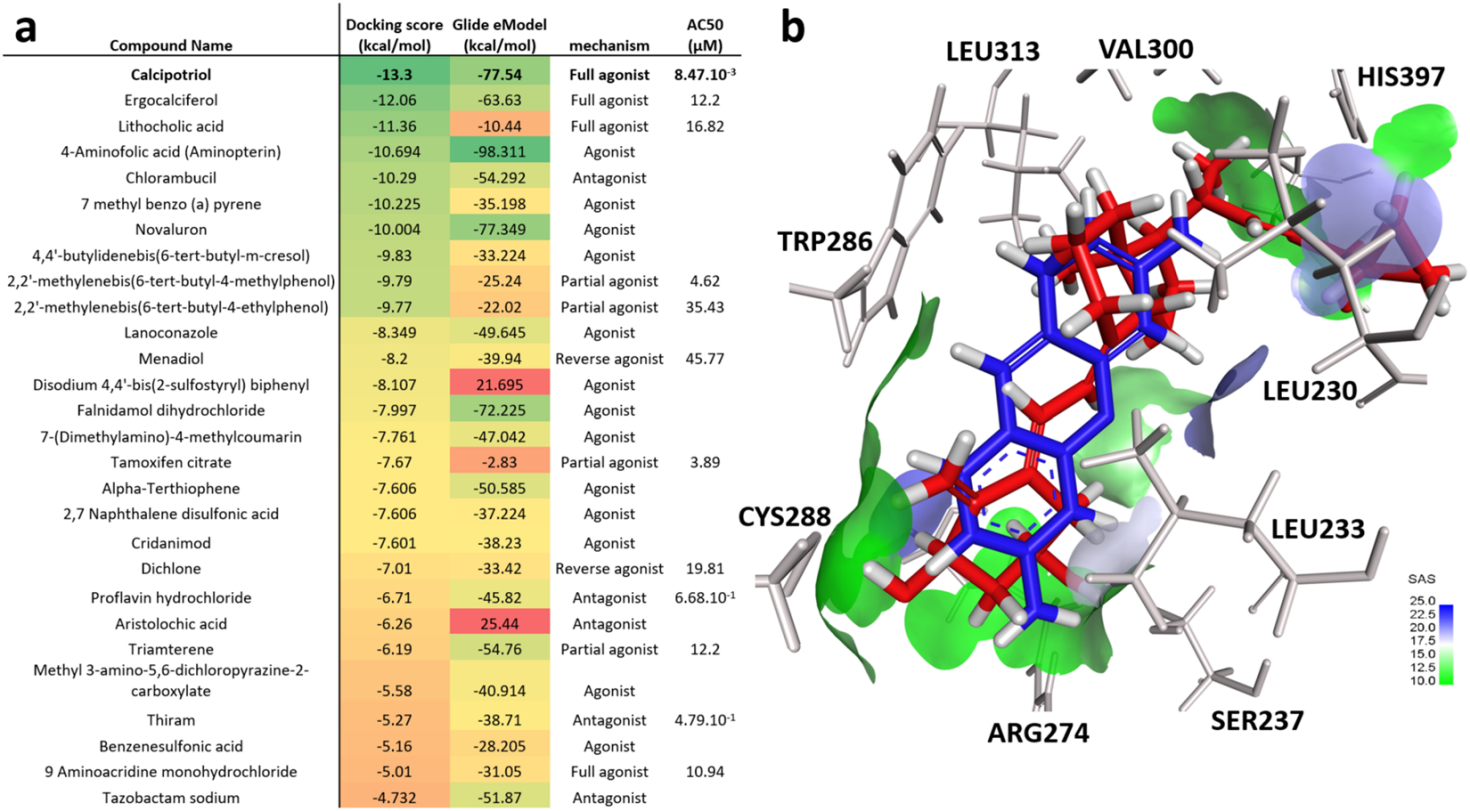
Structure-based molecular docking using Glide and the human VDR structure (PDB code 1S19): (**a**) Docking results for all compounds with their associated XP docking and eModel scores, mechanism and experimental AC_50_ values; (**b**) Binding modes of calcipotriol (red) and proflavine hydrochloride (blue) superimposed in the binding site.

After the structural preparation and cleaning of the VDR structure using the Schrodinger suite^21^ (see Methods), we used Glide^22^ to dock all our selected compounds in the VDR active site. The XP scoring^23^ function was utilized to score the intermolecular interactions between VDR and each compound docked in its site. Compounds’ docking scores (expressed in kcal/mol) and eModel scores (also in kcal/mol) are reported in Fig. 4a. The compound affording the best docking scores (the lower the better) is indeed calcipotriol: in the procedure of self-docking (*i.e*., removal of the native ligand from the crystal followed by its blind re-docking into the active site), the binding conformation of calcipotriol was characterized by an excellent docking score as low as −13.3 kcal/mol and eModel equal to −77.54 kcal/mol (these score levels are typical for nanomolar binders). Moreover, the best predicted docking pose of calcipotriol was found to be reasonably close to the native ligand with RMSD = 2.04Å. This result validated the reliability of the modeling calculations and increased our confidence in the docking results for the other selected compounds. Overall, the molecular docking procedure was able to retrieve as active (docking score < −7 kcal/mol) most of the agonists present in our dataset (encompassing full and partial agonists). However, the docking procedure was not able to discriminate the experimentally confirmed antagonists as active (best docking score obtained for proflavine hydrochloride equal to −6.71 kcal/mol). Interestingly, the molecular docking procedure predicted both compounds (best agonist calcipotriol and best antagonist proflavine hydrochloride) to share almost the same binding pocket (Fig. 4b). The two other non-metal antagonists (aristolochic acid and proscillaridin) were not predicted to fit and bind in the VDR binding pocket we used for our docking experiment.

To refine our results, we performed molecular dynamics simulations (MDS – see Methods for computational protocols) to study the dynamic interactions of the ligands with VDR simulated over 20 nanoseconds. We chose to run a simulation with the full agonist calcipotriol and another with the antagonist proflavine hydrochloride. For both simulations, the best predicted binding pose obtained from the molecular docking calculations was used as the starting binding conformation. Interestingly, results of MDS demonstrated that those binding modes are mainly conserved over the entire simulation, underlining the dynamic stability of these two VDR-ligand complexes. Regarding calcipotriol, the predicted binding mode obtained from molecular docking involved the creation of two hydrogen bonds with the VDR receptor (Supplementary Fig. S1a): one H-bond between His397 and the terminal hydroxyl group, and another H-bond between Ser237 and the methylidene cyclohexanediol. Interestingly, MDS calculations were able to reveal other critical interactions that are likely to play a role in stabilizing calcipotriol in VDR binding site (Supplementary Fig. S1b): one H-bond between His305 and the hydroxyl of the terminal 1-cyclopropylmethanol group, another H-bond between Arg274 and the methylidenecyclohexanediol and two other H-bonds between Tyr143, Ser278 and the other hydroxyl group of the methylidenecyclohexanediol of calcipotriol. The persistence (or conservation ratio) of each of those H-bonds was computed as well: for instance, the H-bond with Ser237 was detected in 96% of the 20,000 MDS frames (*i.e*., one frame every picosecond). Meanwhile, H-bonds with Tyr143 and Ser278 only appeared in 35% and 45% of the MDS frames respectively. Importantly, we observed that H-bonds with His397 and His305 (58% and 54%) were switching from one to the other over the simulation. Meanwhile, the predicted binding mode of proflavine presented a π-π stacking between Trp286 and the aromatic acridine group, as well as one H-bond between Ser237 and one primary amine group (Supplementary Fig. S2a-b). Multiple additional interactions were found through MDS calculations, including H-bonds with Ser278, Ser275, His397, and His305. A π-π stacking interaction between Tyr295 and the aromatic acridine group was also detected through MDS.

### Protein:protein interaction

**Figure 5:**
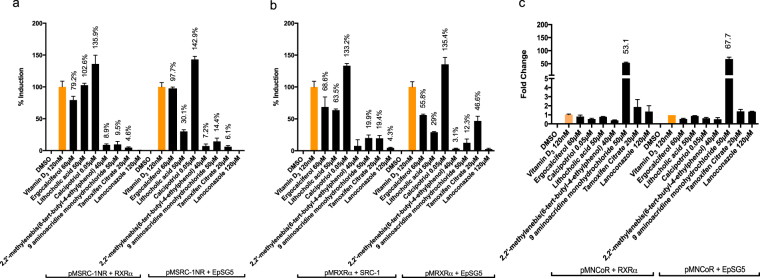
Protein: protein interaction between VDR with RXRα, SRC-1 and NCoR in the presence of select agonists: (**a**) Recruitment of coactivator SRC-1 (SRC/p160 family) by VDR in the presence and absence of RXRα: (**b**) Recruitment of heterodimerization partner RXRα by VDR in the presence and absence of SRC-1 (**c**) Recruitment of corepressors NCoR by VDR in the presence and absence of RXRα. Assays were run in Cos7 cells and data expressed as mean ± SEM (n = 3). Data are normalized to VDR + empty pM vector. Only significant (at least p < 0.05) data points are expressed as a percentage of vitamin D_3_. EpSG5 represents empty pSG5 vector in which RXRα or SRC-1 is expressed.

While transient transfection assays provided us with a global context of chemical receptor transactivation, further functional analysis of ligand induced receptor:coregulator interactions were conducted to gain mechanistic insights into VDR-chemical partnerships. We conducted Mammalian Two Hybrid (M2H) assays on 21 agonists that were previously identified, to examine chemical-stimulated, direct protein:protein interactions between VDR and VDR coregulators. With VDR agonists (see Fig. 5 for representative compounds, Supplementary Table S1**)**, select ligands facilitated the following: 1) Interaction between VDR:RXR and VDR:SRC-1 both in the presence and absence of co-transfected full length RXR and SRC-1. This includes compounds such as calcipotriol, ergocalciferol, LCA, 9 amino acridine monohydrochloride, tamoxifen citrate, triamterene, and 2,2′-methylenebis(6-tert-butyl-4-methylphenol). 2) Interaction between VDR:RXR only in the presence of co transfected SRC-1 was observed with 7 methyl benzo a pyrene and lanoconazole. 3) Interaction between VDR:RXR only in the absence of full length SRC-1 was observed with alpha-terthiophene, 2,2′-methylenebis(6-tert-butyl-4-ethylphenol), and 4,4′-butylidenebis(6-tert-butyl-m-cresol. 4) Interaction between VDR:SRC-1 only in the presence of co-transfected RXR was observed with methyl 3-amino-5,6-dichloropyrazine-2-carboxylate. 5) Interaction between VDR:SRC-1 only in the absence of RXR was demonstrated exclusively by novaluron. 6) There was a grouping of VDR agonists that did not facilitate VDR:RXR or VDR:SRC-1 interactions under any condition including 7-(dimethylamino)-4-methylcoumarin, disodium 4,4′-bis(2-sulfostyryl) biphenyl, 4-aminofolic acid, 2,7 naphthalene disulfonic acid, cridanimod and benzenesulfonic acid. 7) Lastly, except for 9 amino acridine monohydrochloride none of the agonists promoted recruitment of NCoR in the presence or absence of RXR.

**Figure 6:**
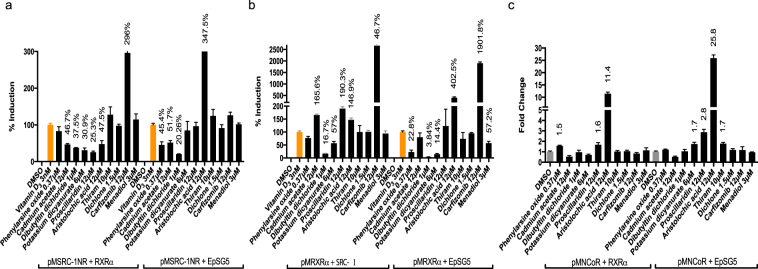
Protein:protein interaction between VDR with RXRα, SRC-1 and NCoR in the presence of select antagonists: (**a**) Recruitment of coactivator SRC-1 (SRC/p160 family) by VDR in the presence and absence of RXRα: (**b**) Recruitment of coregulator RXRα by VDR in the presence and absence of SRC-1 (**c**) Recruitment of corepressor NCoR by select compounds in the presence and absence of RXRα. Note that corepressor recruitment of VDR by antagonists was also tested in the presence of vitamin D_3_, however, values were negligible (data not shown). Assays were run in Cos7 cells and data expressed as mean ± SEM (n = 3). Data are normalized to VDR + empty pM vector. Only significant (at least p < 0.05) data points are expressed as a percentage of vitamin D_3_ for (**a,b**). For (**c**) data was normalized to DMSO set to 1 and no positive control was applied. Only significant values compared to DMSO (at least p < 0.05) are denoted. EpSG5 represents empty pSG5 vector in which RXRα or SRC-1 is expressed.

Comparatively, the potential of antagonists to modify 1,25D_3_ induced VDR:RXR and VDR:SRC-1 interactions was assessed both in the presence and absence of co-transfected full length coregulators (Fig. 6, Supplementary Table S1**)**. The following interactions were observed: 1) Select compounds that consistently attenuate recruitment VDR:RXR and VDR:SRC-1 in both the presence and absence of co-expressed coregulators including dibutyltin dichloride, triphenyltin hydroxide and the cadmium reference solution. 2) Antagonists that selectively inhibit VDR:RXR interactions only in the absence of full length wild type SRC-1 include phenylarsine oxide and menadiol. 3). Antagonists that selectively inhibited VDR:SRC-1 interactions only in the presence of full length wild type RXR include: tributyltin chloride, proscillaridin and potassium dicyanoaurate. 4) Antagonists that selectively inhibited VDR:SRC-1 interactions only in the absence of full length wild type RXR include: cadmium reference solution and phenylarsine oxide. 5) There were no antagonists that selectively attenuated VDR:RXR interactions in the presence of full length SRC-1. Unexpectedly, select VDR antagonists also appeared to enhance some VDR coregulator interactions including: 1) Antagonists that enhanced recruitment of VDR:RXR and VDR:SRC-1 in both the presence and absence of co-expressed coregulators including cadmium dichloride and cadmium dinitrate. 2) Antagonists that enhanced the interaction between VDR:RXR only in the presence of co transfected SRC-1 including proscillaridin and cadmium acetate dehydrate. 3) Antagonists that enhanced the interaction between VDR:SRC-1 only in the presence of co-transfected RXR including carfizomib. 4) Antagonists that enhanced the interaction between VDR:SRC-1 only in the absence of RXR was demonstrated exclusively by aristolochic acid. 5) All antagonists as expected recruited corepressor NCoR both in the presence and absence of RXR.

### Clustering of M2H data

**Figure 7:**
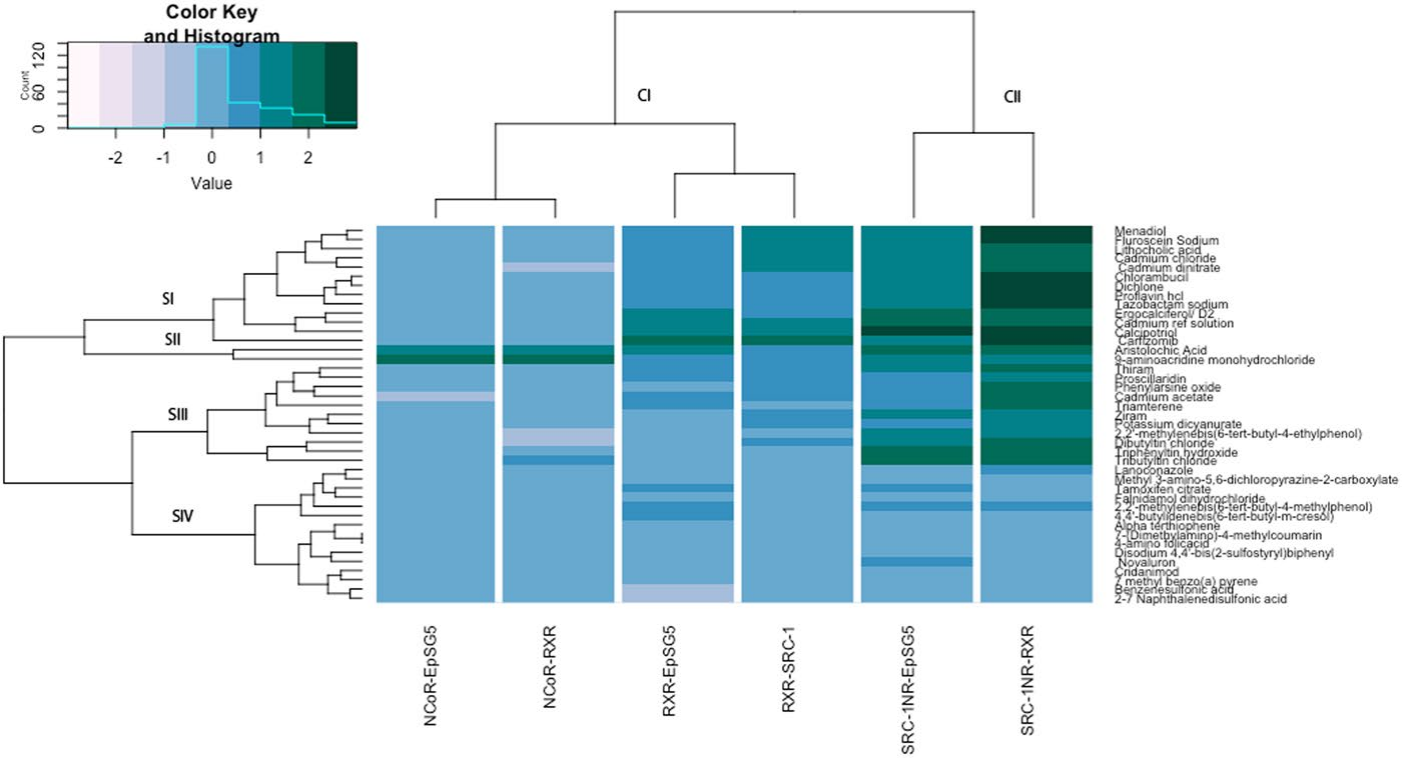
Heat map showing the variability in the selective preference of compounds to enhance or inhibit the ability of VDR to recruit or interact with coregulator (RXRα), coactivator (SRC-1) and corepressor (NCoR-1). Higher recruitment values are indicated in green while lower values are in blue.

In order to visualize VDR functional data in a global context, the mammalian 2-hybrid (M2H) data for all VDR agonists/antagonists tested were visualized as a heat map in Fig. 7. The data resulted in two empirical clusters with CI comprised of VDR:RXR and VDR:NCoR interactions forming a co-cluster and CII comprised of VDR:SRC-1 interactions forming a solitary subcluster. CI is defined by an overall lower level of activity across the majority of VDR agonists/antagonists. CII exhibits an overall higher assay activity across a majority of compounds examined. Within each condition (i.e. RXR, SRC-1 or NCoR) the presence or absences of full length co-regulators to the M2H assay paired together. With NCoR, addition of full length RXR did not appear to significantly facilitate VDR:NCoR interactions. Conversely, addition of full length SRC-1 to VDR:RXR assays and addition of full length RXR to VDR:SRC-1 assays appeared to have an observable effect. In relation to clustering of VDR active compounds, there appeared to be four predominant subclusters. Subcluster SI is comprised of both potent agonists and antagonists and appears to cluster based on both VDR:RXR and VDR:SRC-1 interactions. Subcluster SII is comprised of two compounds that strongly recruited NCoR. Subcluster SIII is predominantly comprised of potent antagonists and a few relatively less potent agonists and is driven through VDR:SRC-1 interactions. Subcluster IV is comprised of VDR agonists that exhibit minimal coregulator recruitment.

### Endogenous CYP24A1 induction

**Figure 8:**
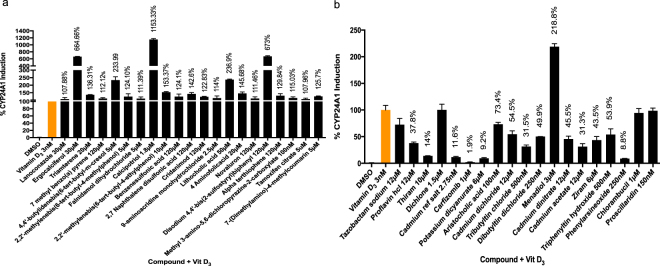
Endogenous CYP24A1 induction in HL-60 cells by compounds in the presence of 3 nM Vitamin D_3_: (**a**) VDR agonists: data expressed as percentage of vitamin D_3_ alone (**b**) VDR antagonists: data are expressed as percentage of vitamin D_3_ alone only for compounds exhibiting significant inhibition of at least p < 0.05.

The ability of VDR agonists/antagonists to induce or inhibit endogenous expression of CYP24A1, a highly inducible transcriptional target of VDR/1,25D_3_ was assessed in human myelocytic leukemic (HL60) cells. To ensure consistency in data outcomes between agonists and antagonists, all assays were conducted in the presence of 3 nM 1,25D_3_ and results were expressed as the percentage of gene induction/repression that surpassed baseline 1,25D_3_ induction alone (Fig. 8a). Concentration of each VDR agonists/antagonist was adjusted to a maximal tolerated dose that did not exhibit HL60 cell cytotoxicity (Supplementary Figures S3-S5). Similar to reporter assays, strong agonists such as calcipotriol, ergocalciferol and lithocholic acid exhibited marked induction compared to weaker agonists such as triamterene, lanoconazole and tamoxifen citrate. However, the induction values of compounds: 4,4′-butylidenebis(6-tert-butyl-m-cresol) and disodium 4,4′-bis(2-sulfostyryl) biphenyl were higher than expected (Fig. 8a). These compounds exhibited weak agonist activity in transient transfection assay, suggesting the possibility of synergistic activities for these compounds. Of the VDR antagonists 14 of 19 significantly inhibited 1,25D_3_ induced expression of CYP24A1 (Fig. 8b). Conversely, the five remaining compounds that exhibited antagonist activity in transactivation studies including tazobactam sodium, aristolochic acid, dichlone, chlorambucil and proscillaridin did not exhibit any marked attenuation below 1,25D_3_ baseline activity in this assay. Among the three potential reverse agonists identified in transient transactivation assays, only menadiol was able to synergize 1,25D_3_ induced CYP24A1 expression in HL60 cells (Fig. 8b).

## Discussion

In this study, Tox21 VDR transactivation data was mined to identify potential modulators of the vitamin D axis from xenobiotic chemicals. We selected a short list of Tox21 compounds for confirmation in orthogonal assays based upon original Tox21 HTS values including compound potency and efficacy, activity in viability assays, curve fit parameters that included the shape of concentration response curve, flags and sensitivity and selectivity of compounds to VDR. The objective for confirmation with orthogonal assays was to address key fundamental questions about nuclear receptor function with VDR as a potential target for xenobiotics. We examined how structurally and functionally diverse compounds may modify (induce or inhibit) core nuclear receptor functions of VDR with respect to its ability to heterodimerize human RXRα, recruit coactivator (SRC-1) and corepressor (NCoR-1) and transactivate the CYP24 promoter. Molecular docking simulations were further conducted to identify key structural interactions between “active” VDR agonists and antagonists and the VDR ligand binding domain.

Transient transactivation assays were performed using full-length human VDR gene reporter construct as opposed to using GAL4-DNA binding domain and NR-ligand binding domain chimeras^14^ to help minimize false negative/positive results. We used a CYP24 promoter fused to a Luciferase reporter in HEK293T cells for this assay. The human CYP24 reporter consists of two DR3 type vitamin D_3_ response elements (VDREs) located between −140 and −300 bp upstream of the transcriptional start site of the human CYP24 gene^24^. Twenty out of 21 agonists were transcriptionally active similar to the qHTS datasets with their activities ranging from an AC_**50**_ of 0.009 μM for calcipotriol to an AC_**50**_ of 37.4 μM for novaluron. As anticipated potent agonists such as calcipotriol exhibit close structural similarities with 1,25D_3_. However, ergocalciferol while still structurally similar to 1,25D_3_ is relatively less potent. Lithocholic acid exhibited an AC_**50**_ of 22.29 μM, which is equivalent to the reported value of 22.39 μM in the VDR beta-lactamase qHTS assay^14^. 9 aminoacridine monohydrochloride, 2,2′-methylenebis(6-tert-butyl-4-ethylphenol) and tamoxifen citrate and lanoconazole were each found to have comparatively weaker transactivational activities with higher AC_**50**_ values. Interestingly, tamoxifen citrate is a potent ER repressor^14^ although it was found to be an active VDR inducer. This again exemplifies the varied nature of xenobiotic interactions with select nuclear receptors that may in fact facilitate differential biological responses depending upon selective receptor interactions and cell specific receptor expression.

The transcriptional repression exhibited by identified VDR antagonists was verified to be the outcome of actual chemical induced VDR inhibition and not artificial interference resulting from cytotoxicity. The use of Dual Glow Luciferase Assay system wherein the expression of an experimental reporter was normalized to that of an internal control reporter which aided in differentiating specific and non-specific cellular responses and offered control over transfection efficiencies between wells (Promega Corp, Madison, WI, USA). Cell viabilities were measured for all cell lines utilized in this study and aided in determining appropriate concentration ranges for test compounds prior to conducting *in vitro* reporter assays. Only concentrations that yielded more than 80% cell viability were chosen to be appropriate (Supplementary Figures S3–5). Antagonists exhibited minimal to marked inhibitory activity with fluorescein sodium as an exception, as it did not exhibit any activity in assays utilized in this study. Being a fluorescent tracer that is used extensively in diagnostic medicine^25^ it is highly likely that was a false positive in the qHTS antagonist assay due to autofluorescence^5^. The remainder of targeted antagonists was confirmed active in the presence of 3 nM 1,25D_3_. Metal containing compounds (10/19) listed here in their increasing order of potencies included phenylarsine oxide, triphenyltin hydroxide, potassium dicyanurate, cadmium reference salt, cadmium acetate, tributyltin chloride, cadmium chloride, dibutyltin dichloride, ziram, and cadmium dinitrate were highly active in repressing VDR transactivation with AC_**50**_ values that ranged between 0.01 μM–1.33 μM (See Table 1). Among these the inhibitory effect of organotins such as the tributyltin chloride and triphenyltin hydroxide, cadmium salts and arsenic-containing compounds are of particular interest, due to well-established linkages to endocrine disruption via activities with other nuclear receptors including PPARy and ER. An important distinction in the effects exist however, in that all three metals tend to have a stimulatory effect on PPARy and ER^26–29^, while they exhibit a potent inhibitory effect on VDR. Nevertheless, functional disruption of vital endocrine receptors including VDR can result in widespread systemic adverse effects. Non-metal containing compounds including chlorambucil, tazobactam sodium, thiram, proflavine hydrochloride, aristolochic acid and proscillaridin additionally had potent inhibitory activity values in the increasing order of listing (AC_**50**_ = 0.02 μM–AC_**50**_ = 1.89 μM). A unique group of reported Tox21 antagonists including carfizomib, dichlone and menadiol did not demonstrate any activity when run in agonist mode. Rather this group of compounds demonstrated a synergistic response in the presence of 1,25D_3_. Carfizomib is a second-generation irreversible (26S) proteasome inhibitor used as a chemotherapeutic agent against multiple myeloma^30^. Proteasomes are responsible for protein degradation including nuclear receptors^31^ and studies have shown that inhibition of proteasome activity can result in increased accumulation and transactivation of nuclear receptors. In fact, Kongsbak^32^ and colleagues (2014) have demonstrated up-regulation of VDR protein expression and increased 1,25D_3_ induced gene activation following proteasome inhibition suggesting that a similar mechanism might be at play with respect to the actions of carfizomib. Dichlone is a potent fungicide and pesticide that also induces global DNA hypomethylation by repressing the action of DNA methyltransferases (DNMTs) suggesting a putative epigenetic role in promoting VDR transactivation^33^. Water-soluble vitamin K3 or menadiol is used to treat coagulopathies associated with obstructive liver disease^34^. It is unclear how this compound induces VDR transactivation and as such its mode of action could at best be speculated.

Moreover, we applied structure-based 3D docking and molecular dynamics simulations to predict and analyze the binding modes of our experimental hits (including both agonists and antagonists). The excellent docking scores afforded by all agonists demonstrated the relevance and ability of our analysis to discriminate those active compounds, similar to other studies that have coordinated molecular docking and cell based functional assays to assess VDR activities^35^. However, our docking model was insufficient to specifically identify VDR antagonists and thus, further investigation using ensemble docking (*i.e*., combinatorial docking using diverse series of conformations for antagonists as well as a collection of different conformations for the VDR binding site) will be necessary to better account for the flexibility of the binding site. We were also unable to correctly dock metal containing compounds, which is also a known limitation of current scoring functions being not well calibrated with metal-containing/bound substances. We then conducted molecular dynamic simulations on calcipotriol (agonist) and proflavine hydrochloride (antagonist) to have a better understanding of the dynamic non-covalent interactions of these two compounds once enclosed in the VDR ligand-binding site. Such analysis was highly critical to determine the relative importance of each residue involved in those interactions. In particular, we demonstrated that the H-bond interaction between calcipotriol and Ser237 played a major role in the binding abilities of the small molecule ligand, as shown by the high conservation ratio through the MD simulation. In fact, Ser237 could be a suitable candidate for a mutagenesis study. Moreover, both Tyr143 and Ser278 also represent important anchors for calcipotriol but presented lower persistence rates through MDS. With proflavine, MDS results demonstrated that this antagonist forms less stable interactions compared to calcipotriol, as illustrated by the interaction persistence scores (all being lower than 40%) and further confirmed by the higher docking scores and the lower experimental potency. However, molecular dynamic simulations and the superimposition of proflavine and calcipotriol also indicated several shared amino acid contact residues and structural arrangement for both compounds within the VDR LBD. The consistency of orientation between these selected compounds suggest that both VDR agonists and non-metal-containing antagonists are capable of dynamic interactions within the receptor, but likely facilitate differential allosteric conformations essential for receptor activation and repression. Interestingly, a recent analysis based on zebrafish VDRα^36^ demonstrated that VDR also presents an alternative-binding site when co-crystalized with the agonist lithocholic acid. The structure used in our analysis remarkably presented this alternative binding pocket. We decided to evaluate the root mean square fluctuation of the amino acids involved in the second binding site (Ser235, Gln239, Asp149 and Lys240) using MDS in presence of calcipotriol and proflavine hydrochloride. Interestingly, the four amino acids presented smaller fluctuations when VDR was interacting with the agonist calcipotriol (average of RMSF = 0.47Å) than with the antagonist proflavine (average of RMSF = 0.68Å). Our MDS results might indicate a tendency of VDR to present a second binding site when interacting with an agonist, but longer in depth MDS computations (up to 10 µs) are needed to validate this hypothesis.

The mammalian 2-hybrid (M2H) assay is a robust tool for studying protein-protein interactions between structural domains or full-length nuclear receptors and other proteins associated with transactivation^37^. Data outcomes from M2H experiments in this study suggest significantly diverse and complex ligand induced protein:protein interactions with VDR and VDR coregulators. Results are consistent with the observation that the holo conformation of VDR is ligand-specific and is pivotal for revealing receptor:coregulator interaction domains associated with RXR heterodimerization and recruitment on coactivators and corepressors^38^. Ligand binding of VDR induces an allosteric shift in receptor conformation, where H12 (AF2 domain) rotates and packs tightly over helices H3, H4, and H5, creating a hydrophobic ligand-binding pocket^15^. The repositioning of H12 creates a “charge clamp” between the negatively charged glutamate residue (E420 in human VDR) of the AF2 region of H12, and positively charged lysine residue (K246 in human VDR) of H3. The charge clamp is responsible for coactivator interaction by directly binding with the LXXLL amino acid motif(s) within the NR box of coactivators^39^. Small changes in ligand structure appear to affect receptor configurations impacting co-activator binding interface and ultimately varying efficacy and potency of NR transactivation. Supporting this model, our data with calcipotriol and ergocalciferol, both agonists in transactivation assays, exhibit the ability to fully recruit both VDR:RXR and VDR:SRC-1 interactions similar to 1,25D_3_. Comparatively, LCA which functions as a less potent VDR agonist, exhibits attenuated recruitment of coregulators compared to 1,25D_3_. Previous studies demonstrate that LCA exhibits a selective pattern of ligand-VDR coregulator associations that distinguishes 1,25D_3_-VDR endocrine from LCA-VDR metabolic functionalities^38^. Similarly, studies examining 1,25D_3_ analogs with select 22-alkyl sidechain substitutes also illustrate that slight modifications to ligand structural result in significant alterations in VDR functionality through altering allosteric receptor interactions within helix 12^40^. The authors of this study provide select models of VDR conformations that distinguish between full VDR agonists, partial VDR agonists and VDR antagonists.

In regards to the functionality of partial VDR agonists, multiple modes of activity have been proposed. For instance, ligand-mediated phosphorylation within the AF1 domain can induce receptor transactivation as observed with selective estrogen receptor modulators^41^. With PPARγ, partial agonists have been demonstrated to facilitate suboptimal positioning of receptor conformations resulting in destabilization of helix H12 distinct from conformations induced with full receptor agonists or antagonists^42^. Partial agonist activity may also result from mixed receptor confirmations where ligands possess both agonist and antagonist properties as previously described for VDR^39,43^. In this study, partial VDR agonists (Supplementary Table 2) predominantly exhibited an attenuated recruitment of VDR:RXR and VDR:SRC-1 interactions in the absence of co-transfection with coregulators compared to 1,25D_3_. This is likely due to an inability of these agonists to re-localize helices H12 and induce a structural transition that triggers the mousetrap-like mechanism stabilizing ligand binding and co-regulator recruitment^44^. However, further studies will be needed to identify exact mechanism(s) for each compound tested.

Supplementation of coregulators facilitated selective alterations in receptor transactivation, and protein:protein interaction between VDR:RXR and VDR:SRC-1 with selected full/partial agonists (See Fig. 5a-c). We have previously demonstrated that VDR co-transfections with full-length coregulators enhances protein:protein interactions between VDR, RXR and SRC-1 with 1,25D_3_ as a primary ligand^45^. Thompson^46^ *et al*., 2001 proposed that the AF2 regions of both VDR and RXR interact with different LXXLL motifs within a single SRC/p160 coactivator. This “bridging” effect of SRC-1 and putatively other coactivators may facilitate stabilization of H12 with less optimal heterodimers. Similarly, both LXXLL motifs of DRIP1 appear to be used by the VDR-RXR heterodimer, suggesting that DRIP1 interacted with the AF2 regions of both receptors^47^. Conversely, VDR:RXR heterodimers may exhibit ligand specific protein recruitment with distinct and separate coactivators. For instance, it has been demonstrated that TIF1 can interact with both RXR and VDR, while SUG1 exclusively interacts with VDR^48^. Differential coactivator recruitment between heterodimer partners may potentially explain our mammalian 2-hybrid data with select ligands. The fact that the cotransfection of either RXR or SRC-1 promotes recruitment between VDR and RXR or SRC-1 suggests the possibility that coregulators enhance stabilization of H12 through bridging or differential recruitment of p160 family members, which can enhance receptor transactivation. One notable exception however was our observation that the EGFR agonist falnidamol hydrochloride induced recruitment of RXR both in the presence and absence of co-transfected SRC-1 although it remained inactive in transient transactivation assay.

In comparison, we anticipated that VDR antagonists would attenuate 1,25D_3_ mediated RXR heterodimerization and recruitment of SRC-1. While a reduction in RXR and SRC-1 recruitment was observed with the majority of antagonists as anticipated, other antagonists enhanced select VDR:coregulator interactions. Notable among such interactions were those of carfizomib, aristolochic acid and cadmium compounds. Each demonstrate the ability to facilitate enhanced recruitment of RXR beyond that of baseline 1,25D_3_ (3 nM) in both the presence and absence of cotransfected SRC-1. Enhanced recruitment of SRC-1 occurred in the presence and absence of cotransfected RXRα with carfizomib and cadmium compounds. Enhanced recruitment of SRC-1 with aristolochic acid occurred only in the absence of cotransfected RXR. Lastly, all antagonists as expected were capable of recruiting NCoR.

Receptor antagonists can be categorized as either “active” or “passive”^49^. Active antagonists tend to have bulky structures that destabilize the active confirmation of helix 12 resulting in stearic obstruction of motifs essential for NR-coactivator interactions. Comparatively, passive antagonists tend to fit into the ligand-binding pocket but facilitate repositioning of H12 to a stable but non-active configuration. A third mechanism of receptor antagonism has also been proposed where antagonists can facilitate H12 stabilization but destabilize other regions of the receptor including the dimerization interface, impeding the ability to form productive heterodimers with RXR^50^. It is worth noting however that non-competitive VDR antagonists have also been identified that function through disruption of VDR-coregulator interactions^51^. The fact that several of the non-metal containing VDR antagonists identified in this study did not afford a good docking score or were not docked at all, may indicate that several of these compounds function through non-ligand mediated mechanisms that disrupt co-regulator interaction ultimately attenuating or inhibiting VDR transactivation.

We next confirmed the effect outcomes of compounds obtained from transient transactivation assay by endogenous gene activation followed by qPCR in human myelocytic leukemic (HL60) cells, a cell line that expresses VDR and effectively induces CYP24A1 expression in the presence of 1,25D_3_^52,53^. Because this cell line proved to be highly sensitive with relatively lower cell viabilities at comparable concentrations used for reporter gene assays, the final concentrations for the majority of compounds were adjusted to prevent cytotoxicity and accommodate healthy cell viability values (>80%). Agonists were tested in the presence of 3 nM of 1,25D_3_ such that the combined fold induction surpassed the relative 1,25D_3_ induction and was expressed as a percentage (Fig. 6a). Expectedly, all agonists further enhanced CYP24A1 expression including falnidamol hydrochloride. While falnidamol hydrochloride failed to transactivate VDR in HEK293T cells, induction of CYP24A1 and recruitment of RXR illustrates that this compound retains VDR functionality that may be cell dependent. However, this needs further investigation. Despite weaker transactivation activities, the induction exhibited by 4,4′-butylidenebis(6-tert-butyl-m-cresol and Disodium 4,4′-bis(2-sulfostyryl) biphenyl could be the result of a synergistic response in the presence of 1,25D_3_. Most antagonists significantly repressed endogenous CYP24A1 expression. However, tazobactam sodium, aristolochic acid, dichlone, chlorambucil and proscillaridin did not follow a similar pattern which could be attributed to the adjusted low doses used for this assay as mentioned earlier. Carfizomib being a potent protease inhibitor and antineoplastic agent effective against multiple myeloma^30^ was predicted to have an inhibitory effect even at low dose (1 µM) in HL60 cells despite its reverse agonist response in other cell types and assays. These variations in the CYP24A1 response not only confirm the high sensitivity of this particular cell line to xenobiotic exposure but also provide further evidence to support the role of VDR as a potential target for xenobiotics that is able to bind structurally diverse endogenous and exogenous compounds and modulate activity of other important genes accordingly.

## Conclusion

Quantitative high throughput chemical screens have been instrumental in identifying compounds that are active towards a variety of nuclear receptors. Those experimental bioprofiles have provided a convenient method of gaining novel information on hundreds of compounds that are potentially toxic, and provide global assessment of ligand interactions with nuclear receptors. In line with the continued surge in scientific interest in dissecting the roles played by NR’s specifically VDR in mechanisms associated with toxicity, we explored the utility of confirming high throughput analysis with orthogonal assays and the potential of VDR as a target of xenobiotics and endocrine disruptors. Through application of *in vitro* cell based assays and *in silico* modeling approaches, we demonstrate the molecular complexity of VDR:ligand interactions and confirm the ability of diverse ligands to modulate VDR, facilitate differential coregulator recruitment and activate/repress receptor-mediated transcription. Further we illustrate that in addition to receptor transactivation, orthogonal *in vitro* assays such as mammalian two-hybrid, and endogenous gene in conjunction with molecular modeling may facilitate a greater understanding of the mechanistic complexities of NR ligands and broad HTS outcomes.

## Methods

### Tox21 chemical library

The Tox21 10 K compound library (https://cfpub.epa.gov/si/si_public_record_report.cfm?dirEntryId=246691) was compiled by the Environmental Protection Agency (EPA), the National Toxicology Program (NTP), and the NIH Chemical Genomics Center/ National Center for Advancing Translational Sciences^14^. It consists of approximately 8,300 unique samples including drugs, food additives, environmental chemicals, consumer product ingredients and industrial chemicals. Compound stock preparation was previously described^54^. Briefly, stock solutions were prepared in DMSO, most at 20 mM, followed by a 3-fold serial dilution series in DMSO resulting in 15 concentrations per compound for testing as described below. Analytical QC information for the library is available at: https://tripod.nih.gov/tox21/samples. qHTS of VDR Beta-lactamase reporter gene and cell viability assays. The assay description and methods are available from the Pubchem Open Chemistry Database, bioassay record AID 743241 (https://pubchem.ncbi.nlm.nih.gov/bioassay/743241#section=Top). Briefly, the cell line and the cell culture reagents for the qHTS screening were from Life Technologies (Carlsbad, CA, USA). The GeneBLAzer® VDR UAS-bla HEK 293 T cells stably expressing a VDR-driven beta-lactamase reporter gene under the control of the upstream activator sequence (UAS) was used. The VDR consisted of the human VDR ligand-binding domain fused to the DNA-binding domain from the yeast GAL4 transcription factor that binds the UAS. Cells were cultured in Minimum Essential Medium (MEM) containing 10% heat-inactivated Fetal Bovine Serum (FBS), 1 mM sodium pyruvate, 0.1 mM NEAA, 100 U/ml penicillin, and 100 mg/ml streptomycin. Cells were grown in a humidified incubator at 37 °C with 5% CO_2_ and passaged when ~70–80% confluent. Prior to screening, GeneBLAzer® VDR UAS-bla HEK 293 T Cells were seeded at 2000 cells/5 µL in black-clear bottom 1536-well plates (Greiner Bio-One North America, Monroe, NC - CONFIRM) using an 8-tip dispenser (Multidrop/Thermo Fisher Scientific, Waltham) and incubated for 5 hours (h) at 37 °C and 5% CO_2_. After incubation, the cells received 23 nl of test compounds in DMSO dispensed using a pin tool station (Kalypsys, San Diego, CA) resulting in final concentrations ranging from 10^−9^–10^−4^ M (15 concentrations). In antagonist mode, 1α, 25-Dihydroxy Vitamin D_3_ (3 nM, final concentration) was added immediately after test compound addition. For the agonist mode screen, each assay plate contained 38 wells for a dose-titration from 0.1 mM (1:3 Dilutions) of 1α, 25-Dihydroxy Vitamin D_3_, along with 16 wells of 50 nM and 16 wells of 15 nM 1α, 25-Dihydroxy Vitamin D_3_ as a positive control. For antagonist-mode screen, each assay plate contained 48 wells exposed to DMSO and 3 nM 1α, 25-Dihydroxy Vitamin D_3_ and 16 wells of 92 µM teraoctyl ammonium bromide and 3 nM of 1α, 25-Dihydroxy Vitamin D_3_. DMSO was used as a vehicle control for compounds. After 16 h (37 °C, 5% CO_2_) of exposure, 1 µL CCF_4_ dye was added to each well with a single tip dispenser. Following 2 h of incubation, fluorescence was measured using 405 nM excitation and fluorescent emissions read-outs at 530 nM (channel 1) and 460 nM (channel 2) using an EnVision plate reader (Perkin Elmer, Shelton, CT, USA). The cytotoxicity effects were measured by the addition of 4 µL of CellTiter-Glo (Promega, Madison, WI, USA) reagent, followed by an additional incubation for 30 min at room temperature. Luminescence was measured on a ViewLux plate reader using an exposure time of 15s.

### Analysis of qHTS data

The qHTS data, processed using the tcpl data pipeline (Filer *et al*., 2016), were downloaded from the EPA website (https://www.epa.gov/chemical-research/toxicity-forecaster-toxcasttm-data). Processing of the data in the tcpl pipeline consisted of the following methods. The ratio of the **r**aw plate fluorescence values (channel 2/channel 1) was determined for each well (rval). Ratios were normalized relative to the positive control compounds (agonist mode: 1α, 25-Dihydroxy Vitamin D_3_, 3 nM, 100%) and DMSO-only wells (0%) by the formula Activity = [(rval − bval)/(pval − bval)] × 100 where rval is the ratio measurement for the treated well, pval is the median value of the positive control wells, and bval is the median value of the DMSO-only wells. The concentration-response data were then fitted with three separate models using the tcpl methods^55^. Briefly, these models were a constant model, a Hill model and as gain loss model (the product of two Hill functions with a shared top). Each model fit was compared using an Akaike Information Criterion (AIC) value and the model with the lowest AIC was selected as the winning model. The AC_50_ and maximum efficacy E_max_ were determined from the winning model. The efficacy values were derived from the top asymptote of the corresponding Hill or Gain-Loss model. They represent the maximum response for a given agonist or antagonist (fold induction or fold inhibition respectively).

### Compound acquisition for orthogonal assay screening

Prioritized compounds were either procured under EPA contract EP-D-12-034 from EvoTec (South San Francisco, CA) or purchased (Sigma-Aldrich Corp., St. Louis, MO, USA). All compounds were serially diluted in DMSO with a final testing concentration ranging from 0.01 to 120 μM.

### Cheminformatics

Molecular docking was conducted using PDB code = 1S19 X-ray crystal structure preprocessed and curated using the Schrodinger Suite and the Protein Preparation Wizard^21^ module and the OPLS3 force field. All the missing side chains were generated using Prime^56^ and protein minimization was performed. The molecular docking procedure was performed using Glide software^22,23^ with XP scoring functions with a rigid protein and flexible ligand. The coordinates of the best calcipotriol and proflavine hydrochloride docking pose were subjected to molecular dynamics simulation using DESMOND. Counter-ions were used to neutralize each complex. The whole system was immersed in a periodic TIP3P water orthorhombic box. The molecular dynamics production run was performed for 20 ns. Each recording interval was 1.0 ps for the trajectory and 1.0 for the energy. The NPT ensemble class with a temperature of 300 K and a pressure of 1.01325 bar was used.

### Heat map

Compound activity patterns for all possible combinations of protein-protein interactions between VDR and its coregulator, coactivator, and corepressor and how individual compounds (agonists and antagonists) affect this interaction. Clustering was performed on the mammalian two hybrid data using the hclust function in R with a Manhattan distance metric and complete linkage^57–59^.

### Plasmid DNA constructs

The pSG5-Human VDR construct was a gift from Dr. John Moore (GlaxoS- mithKline, Research Triangle Park, NC). All human coregulator transient transactivation and mammalian 2-hybrid constructs were a gift from Dr. Donald McDonnell (Duke University, Durham, NC). The CYP24 luciferase reporter, 5XGal4-TATA- Luc mammalian 2-hybrid luciferase reporters, and the pRL-CMV (Renilla luciferase) internal luciferase control were obtained as described previously^45,60^.

### Cell culture

Cell culture media and other necessary reagents were obtained from Life technologies (Carlsbad, CA). Hek293T cells were grown in Minimum Essential Medium (MEM) containing 10% heat-inactivated Fetal Bovine Serum (FBS), 1 mM sodium pyruvate, 0.1 mM NEAA, 100 U/ml penicillin, and 100 mg/ ml streptomycin. Cos 7 cells were cultured in Dubelco’s Modified Eagle Medium (DMEM) containing 10% FBS. Human promyelocytic leukemic HL60 cells were grown in RPMI suspension media containing 15% FBS and 200nM L-glutamine. Cells were maintained in a humidified incubator at 37 °C with 5% CO_2_ and passaged when ~70–80% confluent.

### Transient transactivation assay

Full-length VDR constructs were tested in transient transactivation assays with 1α, 25-dihydroxyvitamin D_3_ (1, 25D3) (EMD Millipore, Billerica, MA) as the positive control. Experiments were conducted using pSG5hVDR, pRLCMV, and CYP24-Luc expression vectors as previously described^59^. Hek293T cells were seeded in 96-well plates at 2.5 × 10^4^ cells per well 24 hours prior to transfection. Cells were transfected at 90–95% confluency using Lipofectamine 2000 (Life Technologies, Grand Island, NY) with DNA diluted in Opti-MEM I Reduced Serum Medium as per the manufacture’s recommendations. For functional comparisons, 89.7 ng of each pSG5-VDR construct was transiently transfected into Hek293T cells with 19.2 ng CYP24-Luc and 4.5 ng of *Renilla* luciferase, which serves as an internal luciferase control (Promega Corporation, Madison, WI). The media was changed 18 hours post transfection and cells were exposed to compounds in fresh DMEM media. Twenty-four hours post-exposure to compounds the Dual-Glo Luciferase Assay System (Promega Corporation, Madison, WI) was used to passively lyse the cells and test for luciferase activity following the manufacturer’s protocols. Luciferase activities were measured using a Wallac MicroBet TriLuc Luminometer (Perkin Elmer Life Sciences, Waltham, MA). Control reactions included empty pSG5 vector and ethanol as a vehicle control. Luciferase readings were normalized to the internal *Renilla* control, and VDR response was normalized to an empty vector control. Transient transfection data were used with the ToxCast Analysis Pipeline (TCPL) to generate dose-response curves and estimate AC_50_ values^55,57^. Curve fitting was performed using Hill, Gain Loss (GNLS), and constant fit models. AC_50_ values were generated for antagonists or agonists, respectively, and were chosen from either the Hill or GNLS model using the Akaike Information Criterion.

### Mammalian 2-hybrid assays

Protein protein interactions between VDR and its heterodimer partner RXRα and members of SRC/p160 family of nuclear receptor coactivators and co-repressor (NCoR1) were assessed using a mammalian 2-hybrid system (Clontech, Mountain View, CA). Assays were conducted with chimeric VDRs containing the herpes simplex VP16 activation domain fused to full length human VDR as prey (pVP16-hVDR). NR co-regulators were used as bait for each reaction, and consisted of fusion proteins containing; a complete NR Box of the SRC-1 (pM-SRC1 aa 241–386), NCOR (pM NCoR-1), or full-length hRXRα fused to the yeast Gal4 DNA-binding domain. Assays were conducted in Cos7 cells seeded into 96 well plates twenty-four hours pre-transfection as described above. Cells were transfected with 33.6 ng pVP16-VDR, 33.6 ng pM-coregulator, 126.6 ng 5XGal4-TATA-Luc reporter (containing response elements for the yeast Gal4 DNA binding domain), and 3 ng *Renilla* using Lipofectamine 2000 as described above. Controls consisted of transfections containing empty pM, pVP16 or both empty pM and pVP16 vectors. For both assays, experiments were replicated three times in groups of 3 technical replicate wells. One-way ANOVAs followed by Tukey’s HSD post hoc tests, sigmoidal dose-response calculation with variable slopes followed by nonlinear regression analysis were run in GraphPad Prism version 6 (GraphPad Software, La Jolla, CA). Note that all assays were conducted in either the presence or absence of co-transfected full length RXR or SRC-1 to assess if exogenous protein expression would further facilitate VDR co-regulator/co-activator interactions.

### Cell viability assay

HEK293T, Cos7, and HL-60 cells were seeded in 96 well plates in triplicate at a density of 25,000 cells/well, transfected and dosed with select concentrations of the test compounds and incubated for 18 hours at 37 °C/5% CO_2_. Triton X (0.1%), DMSO (0.1%), and untreated wells served as controls. Resazurin at 1 × concentration (20 µl) /well was added to the cells and incubated at 37 °C for 2 hours in the dark. The amount of resorufin produced proportional to the number of viable cells was quantified by using a microplate reader (described earlier) equipped with a 560 nm excitation/590 nm emission filter set. Only concentrations that yielded more than 80% viable cells were selected for *in vitro* assays.

### Real-time PCR

Total RNA was isolated from treated HL60 cells using the Zymo RNA Isolation kit (Zymo Research Corp, CA USA) and reverse transcribed using a High Capacity cDNA Archive Kit (Applied Biosystems, Foster City, CA) following the manufacturers’ instructions. CYP24A1 mRNA expression was normalized against that of housekeeping gene GAPDH. Real-time PCR assays were performed in 96-well optical plates on an ABI Prism 7300 Sequence Detection System with SYBR Green PCR Master Mix. Primers used for GAPDH mRNA expression were designed as follows: [GAPDH-F: 5′-CGACCACTTTGTCAAGCTCA-3′ GAPDH-R: 5′-GAGGGTCTCTCTCTTCCTCT-3′], while those for CYP24A1 [CYP24A1-F: 5′-TGAACGTTGGCTTCAGGAGAA-3′,CYP24A1-R: 5′-AGGGTGCCTGAGTGTAGCATCT-3′] were adopted from Yosuke^61^ *et al*. 2009. Fold gene induction following treatments were calculated based on the equation: Fold change = 2^−ΔΔCt^, where ΔCt represents the differences in cycle threshold numbers between CYP24A1 and GAPDH, and ΔΔCt represents the relative change in these differences between control and treatment groups^62^. Values were plotted as a percentage and compared to the percentage induction of vitamin D_3_^52^.

### Data availability

All data generated or analyzed during this study are included in the published article (and its Supplementary information files).

## Electronic supplementary material

Supplementary Information
