# Supplementary material for: Confirmation of high-throughput screening data and novel mechanistic insights into VDR-xenobiotic interactions by orthogonal assays
Source: Sci Rep. 2018 Jun 11;8:8883. doi: 10.1038/s41598-018-27055-3 (PMC5995905; doi:10.1038/s41598-018-27055-3)
Supplement: Supplementary file 1 — Supplementary Information [file 41598_2018_27055_MOESM1_ESM.pdf]

Supplementary Data for: Confirmation of High-Throughput screening data and novel mechanistic insights into VDR-xenobiotic interactions by orthogonal assays

Debabrata Mahapatra[1], Jill A. Franzosa[5], Kyle Roell[2], Melaine Agnes Kuenemann[2], Keith A. Houck[5], David M. Reif[2], Denis Fourches[2], Seth W. Kullman[3,4]

[1] Comparative Biomedical Sciences, College of Veterinary Medicine, North Carolina State University, Raleigh, North Carolina, United States of America; [2] Bioinformatics Research Center, North Carolina State University, Raleigh, North Carolina, United States of America; [3] Department of Biological Sciences, North Carolina State University, Raleigh, North Carolina, United States of America, [4] Program in Environmental and Molecular Toxicology, North Carolina State University, Raleigh, North Carolina, United States of America, [5]National Center for Computational Toxicology, Office of Research and Development, U.S. Environmental Protection Agency, RTP, NC.

Supplementary Table S1. Summary of Mammalian 2-hybrid (M2H) data across all compounds. Compounds exhibiting statistically significant recruitment or inhibition of coregulators (RXR)/coactivators (SRC-1) have values expressed as percentages. Note that in addition to most agonists, antagonists do facilitate recruitment (FR) or inhibit recruitment (IR) in the presence or absence of co-transfected coregulators/coactivators. For corepressor (NCoR) recruitment data was expressed as fold induction compared to DMSO. NS indicate non-significant induction/inhibition values for coregulator/coactivator/corepressor recruitment.

**Agonists**	**pMSRC-1 + pVP16 hVDR**				**pMRXR + pVP16 hVDR**				**pMNCoR + pVP16 hVDR**			
**Co Regulator addition**	**+RXR**		**-RXR**		**+SRC-1**		**-SRC-1**		**+RXR**		**-RXR**	
	**Outcome**	**Value**	**Outcome**	**Value**	**Outcome**	**Value**	**Outcome**	**Value**	**Outcome**	**Value**	**Outcome**	**Value**
Vitamin D_3	FR	100	FR	100	FR	100	FR	100		DMSO		DMSO
Calcipotriol	FR	135.9	FR	142.9	FR	133.2	FR	135.4		NS		NS
Lithocholic acid	FR	102.6	FR	30.1	FR	63.5	FR	29		NS		NS
7-(Dimethylamino)-4-methylcoumarin		NS		NS		NS		NS		NS		NS
Disodium 4,4'-bis(2-sulfostyryl) biphenyl		NS		NS		NS		NS		NS		NS
4-Aminofolic acid		NS		NS		NS		NS		NS		NS
Ergocalciferol	FR	79.2	FR	97.7	FR	68.6	FR	56.8		NS		NS
Alpha-Terthiophene		NS		NS		NS	FR	3.5		NS		NS
Triamterene	FR	63.7	FR	0.9	FR	3.7	FR	8.4		NS		NS
Novaluron		NS	FR	0.9		NS		NS		NS		NS
2,2'-methylenebis(6-tert-butyl-4-ethylphenol)	FR	8.9	FR	7.2		NS	FR	3.1		NS		NS
9 Aminoacridine monohydrochloride	FR	9.5	FR	14.4	FR	19.9	FR	12.3	FR	53.1	FR	67.7
2,2'-methylenebis(6-tert-butyl-4-methylphenol)	FR	22.2	FR	8.2	FR	100.7	FR	79.6		NS		NS
4,4'-butylidenebis(6-tert-butyl-m-cresol	FR	0.91	FR	3.6		NS	FR	96.8		NS		NS
Tamoxifen citrate	FR	4.6	FR	6.1	FR	19.4	FR	46.6		NS		NS
Methyl 3-amino-5,6-dichloropyrazine-2-	FR	0.26		NS	FR	4.2	FR	4.4		NS		NS

carboxylate												
2,7 Naphthalene disulfonic acid		NS		NS		NS		NS		NS		NS
Cridanimod		NS		NS		NS		NS		NS		NS
7 methyl benzo (a) pyrene		NS		NS	FR	1.5		NS		NS		NS
Benzenesulfonic acid		NS		NS		NS		NS		NS		NS
Falnidamol dihydrochloride		NS		NS	FR	3.7	FR	5.3		NS		NS
Lanoconazole		NS		NS	FR	4.3		NS		NS		NS
**Antagonists**	**pMSRC-1NR + pVP16 hVDR**				**pMRXR + pVP16 hVDR**				**pMNCoR + pVP16 hVDR**			
**Co Regulator addition**	**+ RXR**		**- RXR**		**+SRC-1**		**-SRC-1**		**+ RXR**		**- RXR**	
	**Outcome**	**Value**	**Outcome**	**Value**	**Outcome**	**Value**	**Outcome**	**Value**	**Outcome**	**Value**	**Outcome**	**Value**
Vitamin D_3	FR	100	FR	100	FR	100	FR	100		DMSO		DMSO
Fluorescein sodium		NS		NS		NS		NS	FR	1.1	FR	1.2
Cadmium chloride	FR	241.9	FR	258	FR	292	FR	175	FR	0.5	FR	1.1
Tributyltin chloride	IR	24.9		NS	IR	16.7	IR	0.5	FR	1.8	FR	2.2
Thiram		NS		NS		NS		NS	FR	1	FR	1.7
Aristolochic acid	IR	47.5	FR	347.5	FR	146.9	FR	402.5	FR	11.4	FR	25.8
Proflavine hydrochloride		NS		NS		NS		NS	FR	0.8	FR	1.3
Tazobactam sodium		NS		NS		NS		NS	FR	0.8	FR	1.7
Carfizomib	FR	296		NS	FR	2663.1	FR	1908.1	FR	0.8	FR	1.1
Phenylarsine oxide		NS	IR	45.4		NS	IR	22.8	FR	1.5	FR	1.2
Proscillaridin	IR	25.3		NS	FR	190		NS	FR	1.6	FR	2.8
Chlorambucil		NS		NS		NS		NS	FR	1	FR	0.9
Cadmium acetate dihydrate	IR	46.7	IR	51.7	FR	165.6		NS	FR	0.5	FR	0.5
Cadmium reference solution	IR	0.9	IR	1.3	IR	16	IR	23.7	FR	0.8	FR	0.7
Dichlone		NS		NS		NS		NS	FR	1	FR	1.1

Menadiol		NS		NS		NS	IR	57.2	FR	1.1	FR	0.9
Potassium dicyanoaurate	IR	30.9		NS	IR	57	IR	14.4	FR	0.7	FR	1.7
Cadmium dinitrate	FR	168.1	FR	233	FR	270	FR	283	FR	0.5	FR	0.6
Dibutyltin dichloride	IR	37.5	IR	20.2	IR	16.7	IR	3.84	FR	0.9	FR	1
Triphenyltin hydroxide	IR	30	IR	55.8	IR	1.8	IR	0.5	FR	1.1	FR	1.5
Ziram	IR	48.3	FR	189	IR	45.1	IR	25.1	FR	0.4	FR	0.9

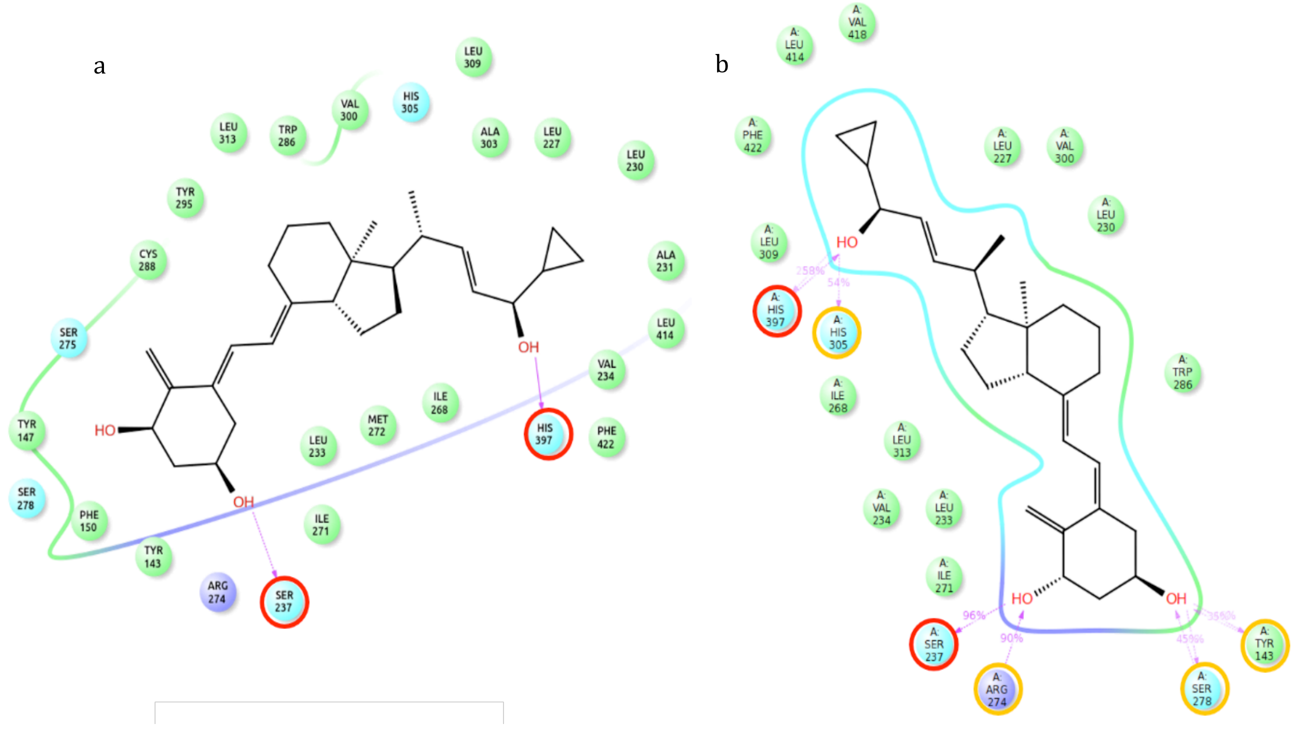

Supplementary Figure S1. a. 2D predicted binding mode of calcilpotriol using molecular docking, b. 2D predicted binding mode of calcilpotriol using molecular dynamic simulations. Highlighted in red the interaction in common between the molecular docking and the molecular dynamic procedures, highlighted in orange the interaction only detected during the molecular dynamic procedure.

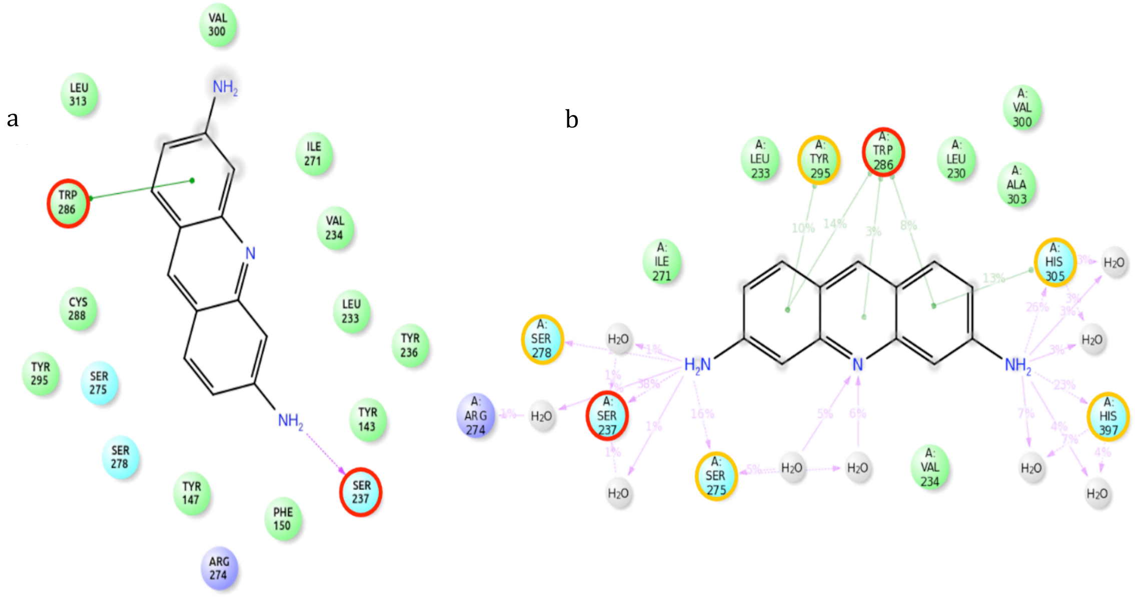

Supplementary Figure S2. a. A 2D predicted binding mode of proflavine hydrochloride using molecular docking, b. A 2D predicted binding mode of proflavine hydrochloride using molecular dynamic simulations. Highlighted in red the interaction in common between the molecular docking and the molecular dynamic procedures, highlighted in orange the interaction only detected during the molecular dynamic procedure.

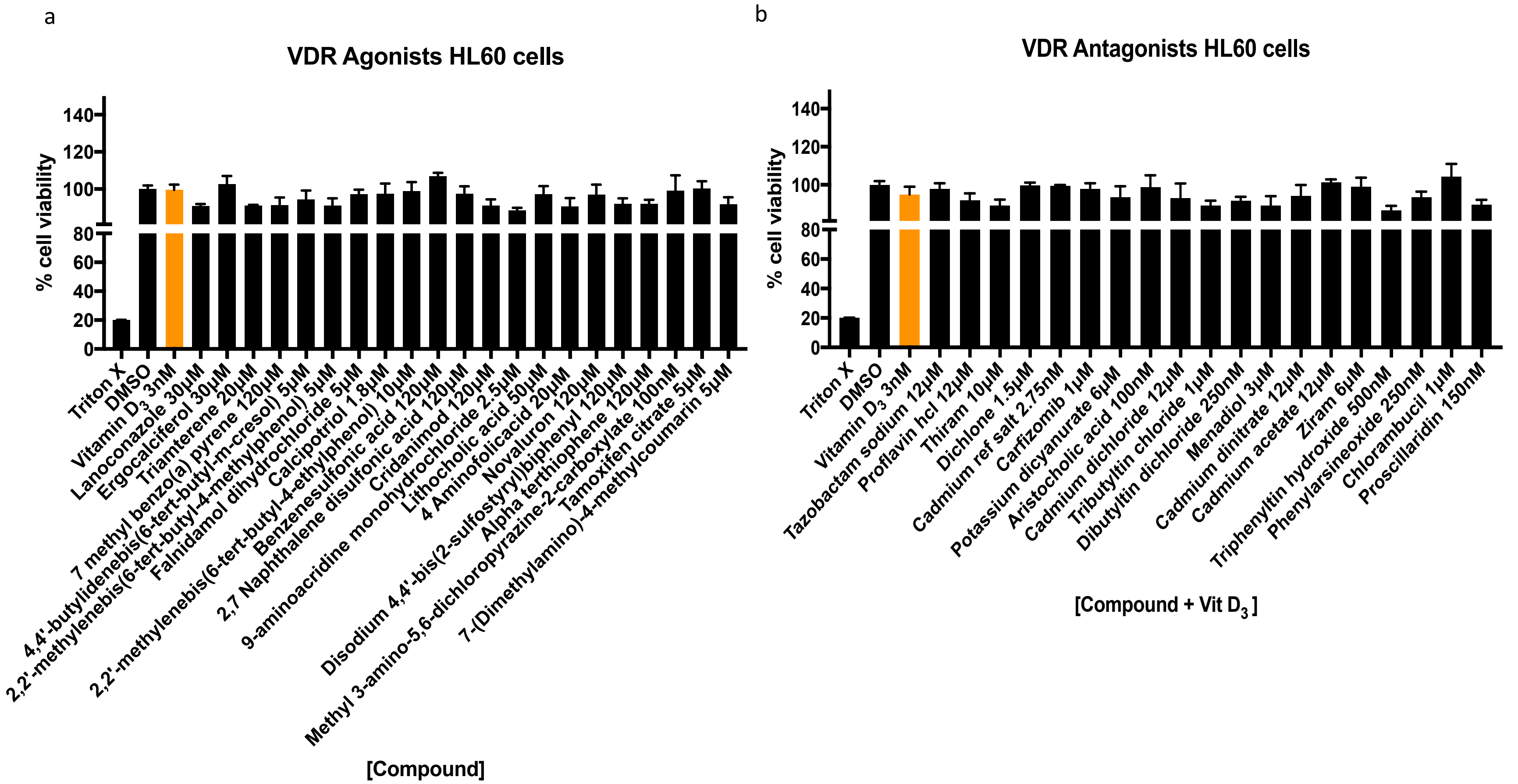

Supplementary Figure S3. Figure showing the results of cell viability assay in HL-60 cells for a. VDR agonists and b. VDR antagonists. The concentrations of compounds corresponding to cell viability percentages of more than 80 were considered appropriate for testing in in-vitro assays. The viability percentage of treatments was measured against that of DMSO. Cells treated with 0.1% Triton X acted as a negative control while those treated with Vitamin D3 acted as a positive control. Data were measured as SEM (n=3) and plotted with GraphPad prism.

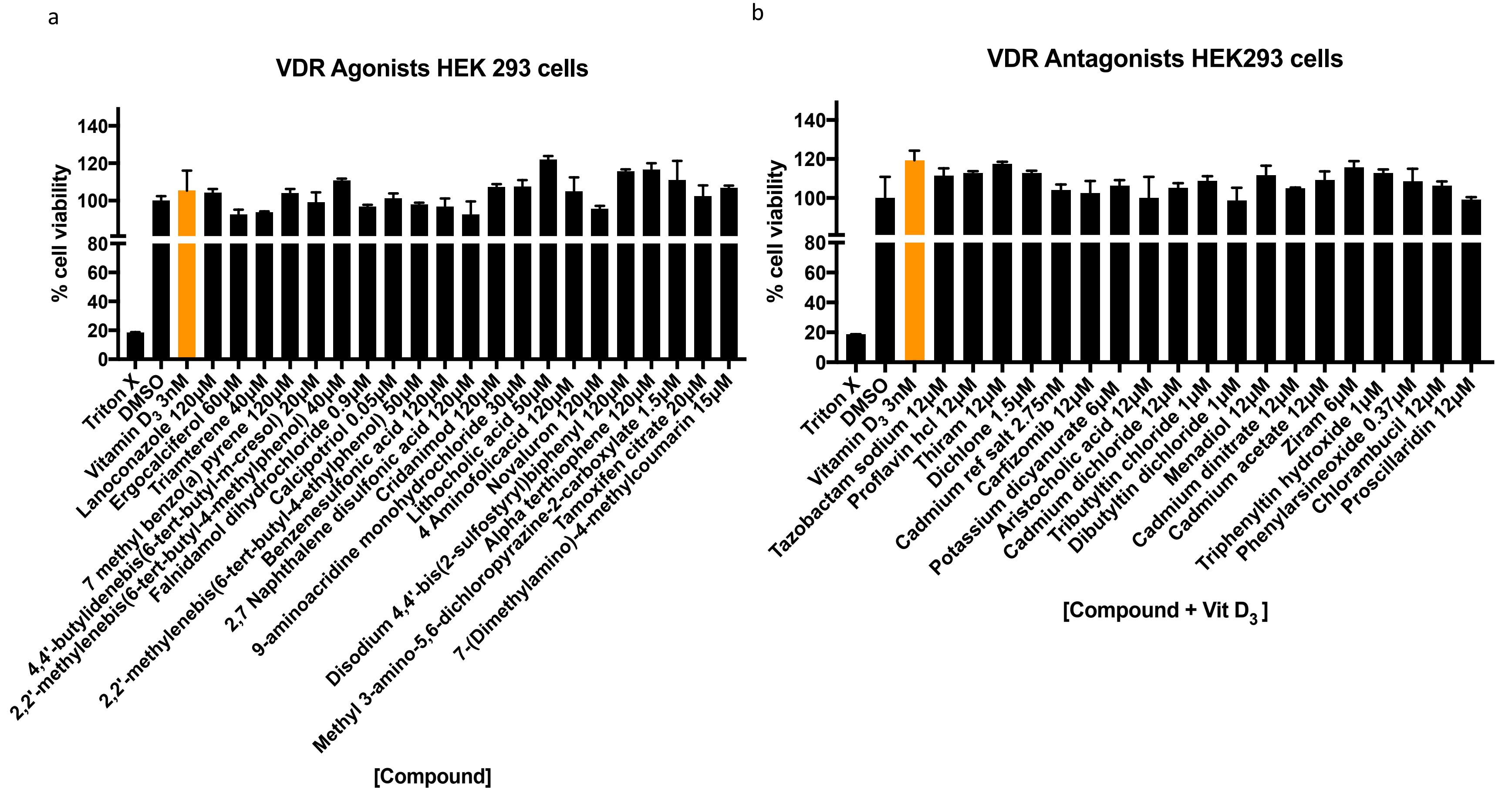

Supplementary Figure S4. Figure showing the results of cell viability assay in HEK293 cells for a. VDR agonists and b. VDR antagonists. The concentrations of compounds corresponding to cell viability percentages of more than 80 were considered appropriate for testing in in-vitro assays. The viability percentage of treatments was measured against that of DMSO. Cells treated with 0.1% Triton X acted as a negative control while those treated with Vitamin D3 acted as a positive control. Data were measured as SEM (n=3) and plotted with GraphPad prism.

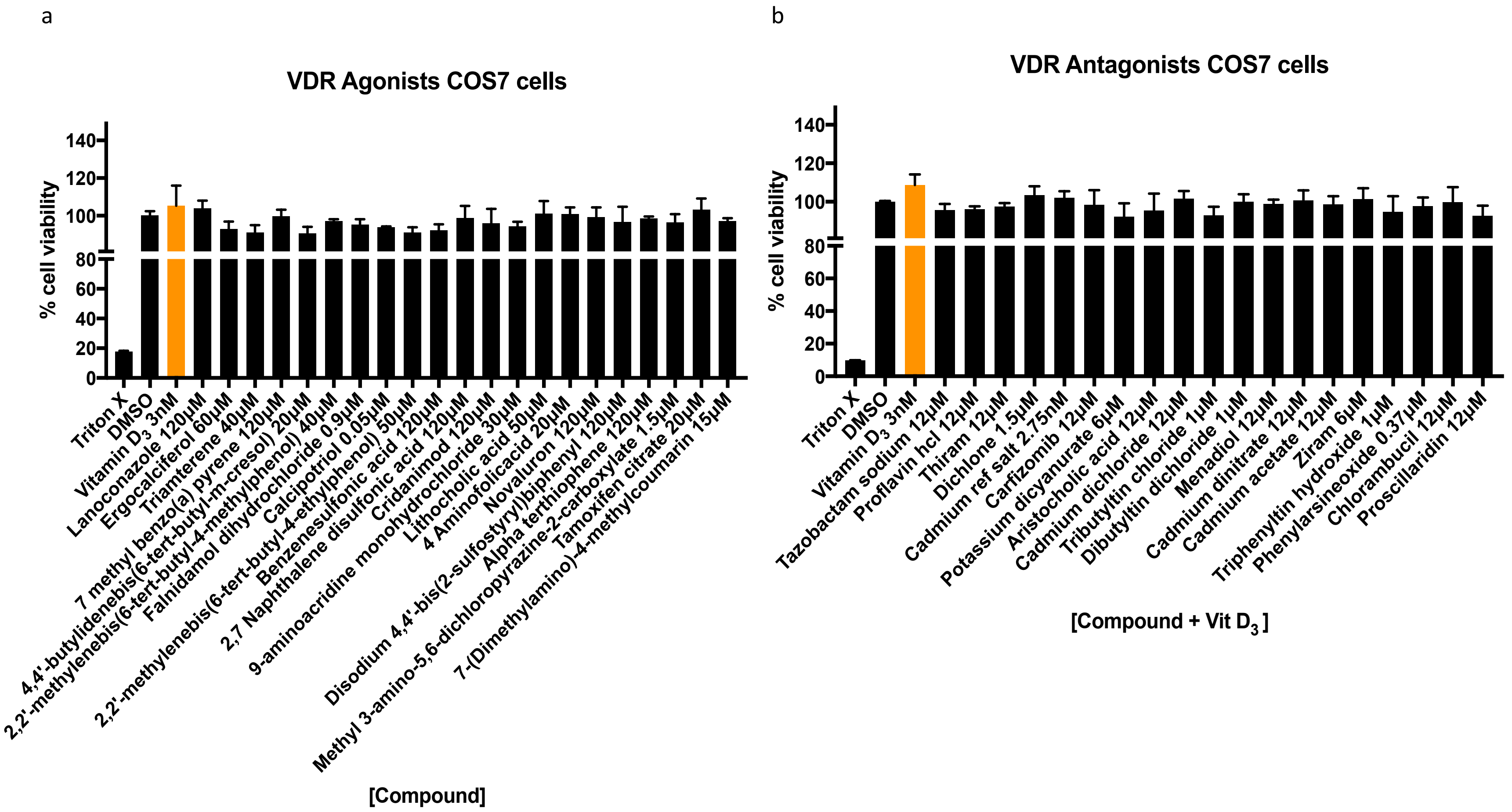

Supplementary Figure S5. Figure showing the results of cell viability assay in Cos7 cells for a. VDR agonists and b. VDR antagonists. The concentrations of compounds corresponding to cell viability percentages of more than 80 were considered appropriate for testing in in-vitro assays. The viability percentage of treatments was measured against that of DMSO. Cells treated with 0.1% Triton X acted as a negative control while those treated with Vitamin D3 acted as a positive control. Data were measured as SEM (n=3) and plotted with GraphPad prism.

VDR agonists

a

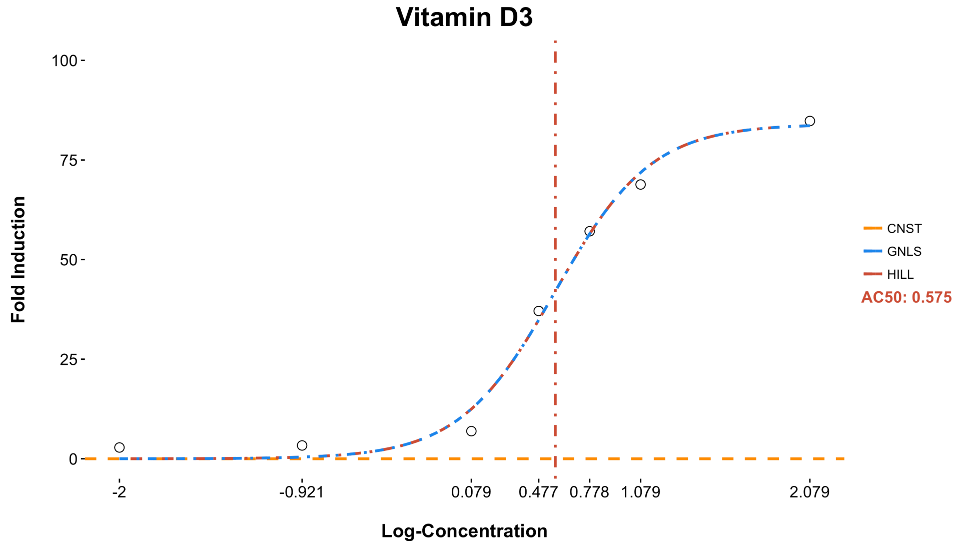

b

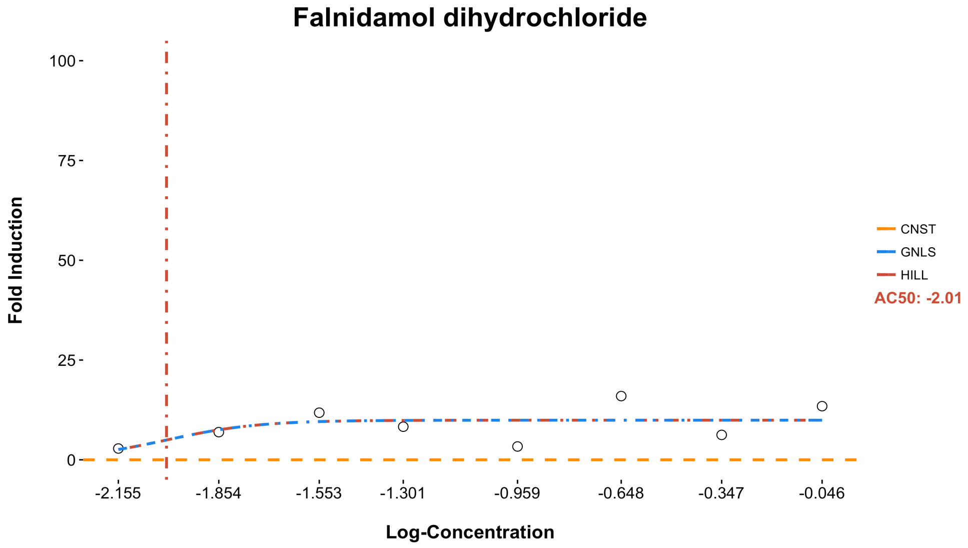

c

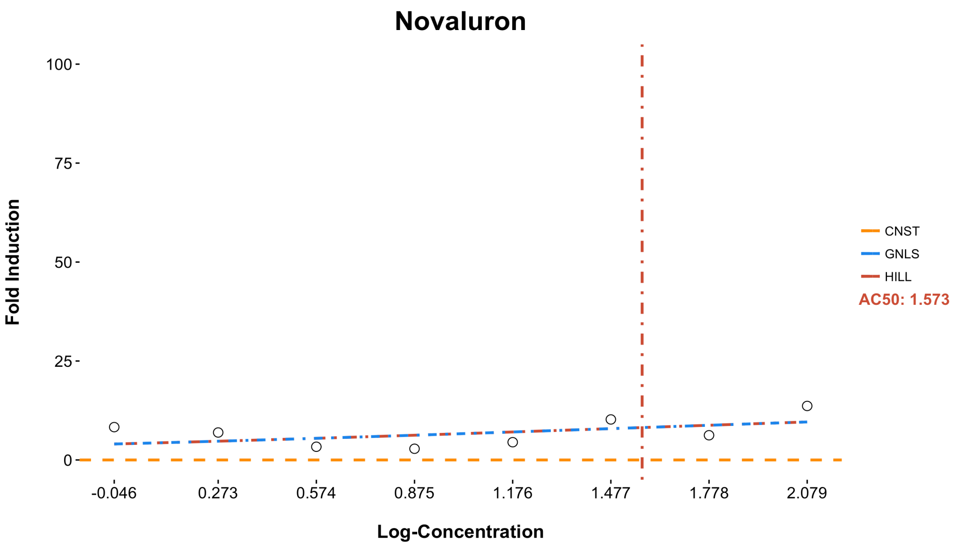

Novaluron
100
75
50
25
0
Fold Induction
-0.046
0.273
0.574
0.875
1.176
1.477
1.778
2.079
Log-Concentration
CNST
GNLS
HILL
AC50: 1.573

d

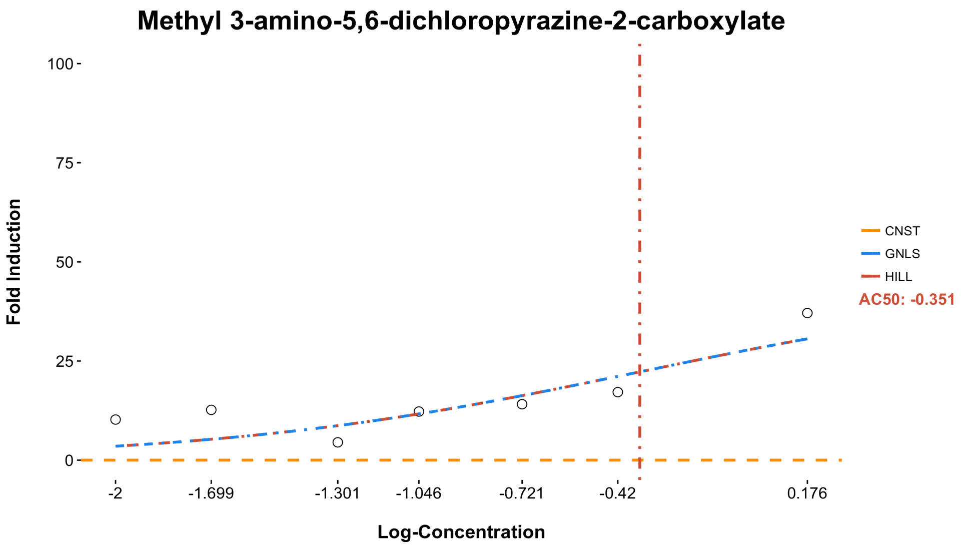

Methyl 3-amino-5,6-dichloropyrazine-2-carboxylate
100
75
50
25
0
Fold Induction
-2
-1.699
-1.301
-1.046
-0.721
-0.42
0.176
Log-Concentration
CNST
GNLS
HILL
AC50: -0.351

e

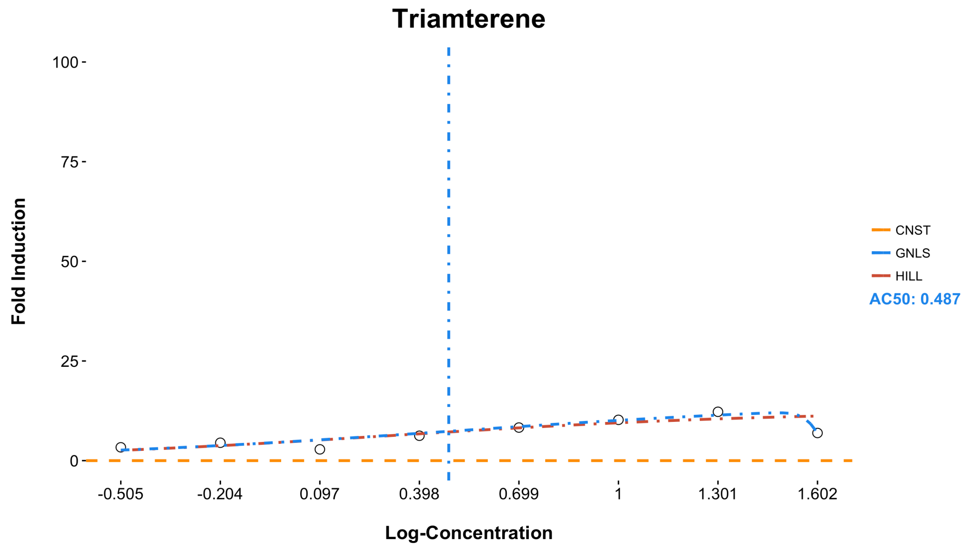
Triamterene
100
75
50
25
0
Fold Induction
-0.505
-0.204
0.097
0.398
0.699
1
1.301
1.602
Log-Concentration
CNST
GNLS
HILL
AC50: 0.487

f

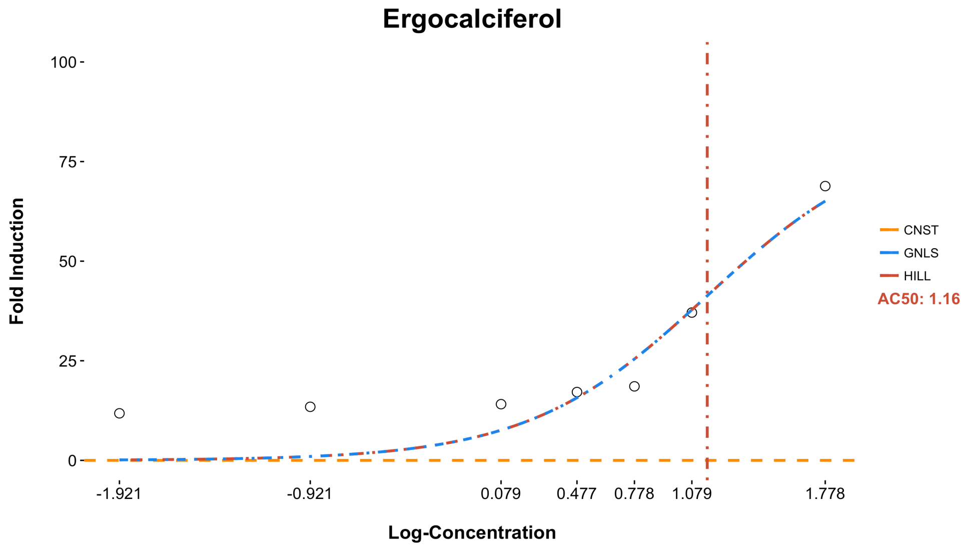
Ergocalciferol
100
75
50
25
0
Fold Induction
-1.921
-0.921
0.079
0.477
0.778
1.079
1.778
Log-Concentration
CNST
GNLS
HILL
AC50: 1.16

g

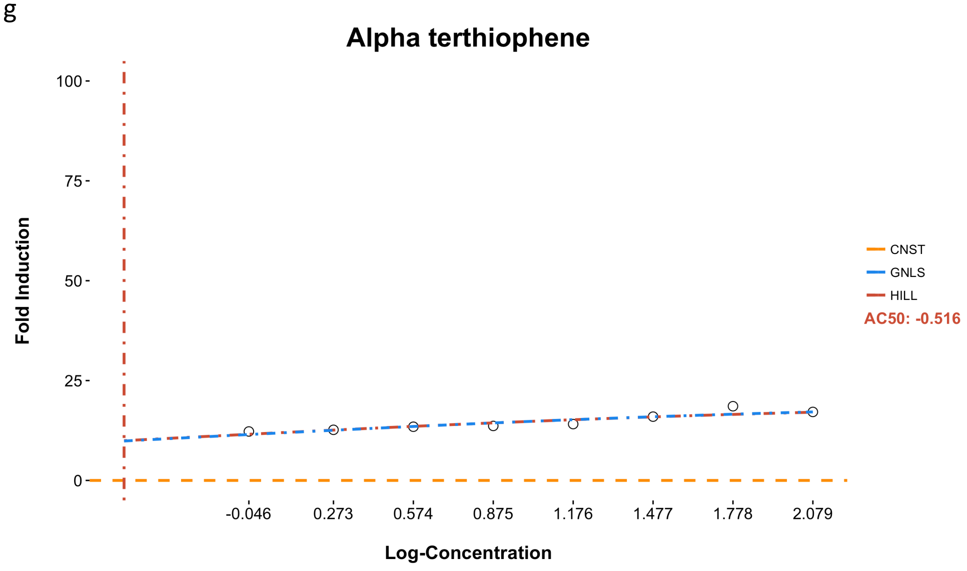
Alpha terthiophene
100
75
50
25
0
Fold Induction
-0.046
0.273
0.574
0.875
1.176
1.477
1.778
2.079
Log-Concentration
CNST
GNLS
HILL
AC50: -0.516

h

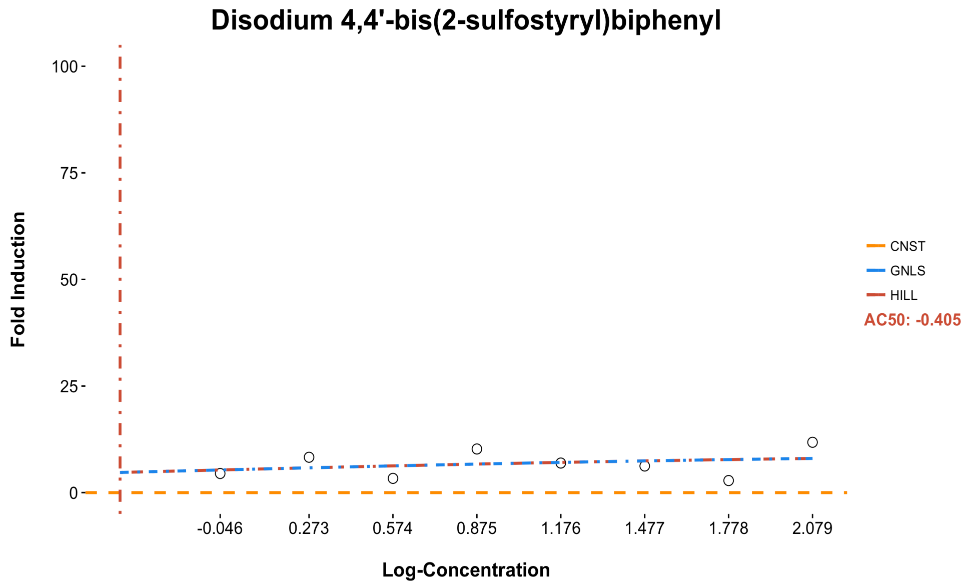
Disodium 4,4'-bis(2-sulfostyryl)biphenyl
100
75
50
25
0
Fold Induction
-0.046
0.273
0.574
0.875
1.176
1.477
1.778
2.079
Log-Concentration
CNST
GNLS
HILL
AC50: -0.405

i

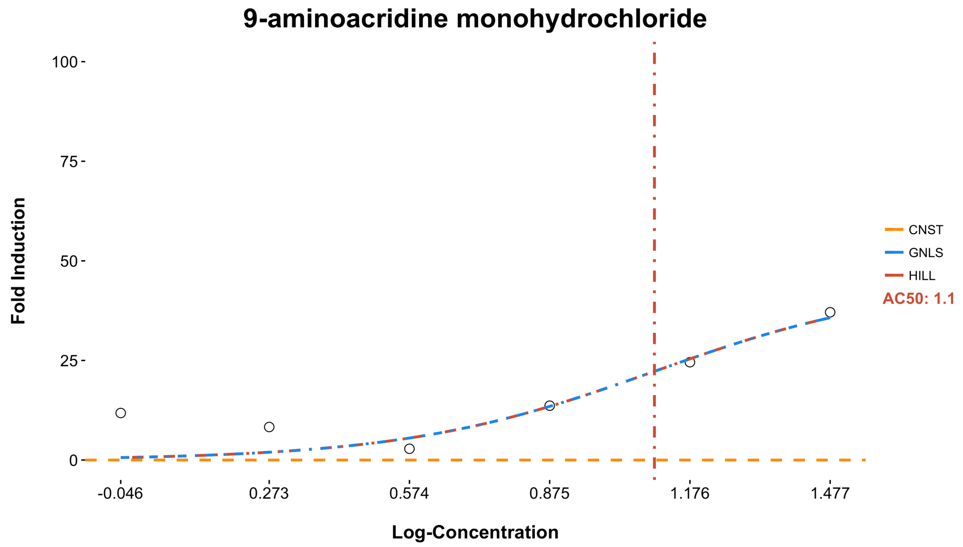

9-aminoacridine monohydrochloride
Fold Induction
100
75
50
25
0
-0.046
0.273
0.574
0.875
1.176
1.477
Log-Concentration
CNST
GNLS
HILL
AC50: 1.1

j

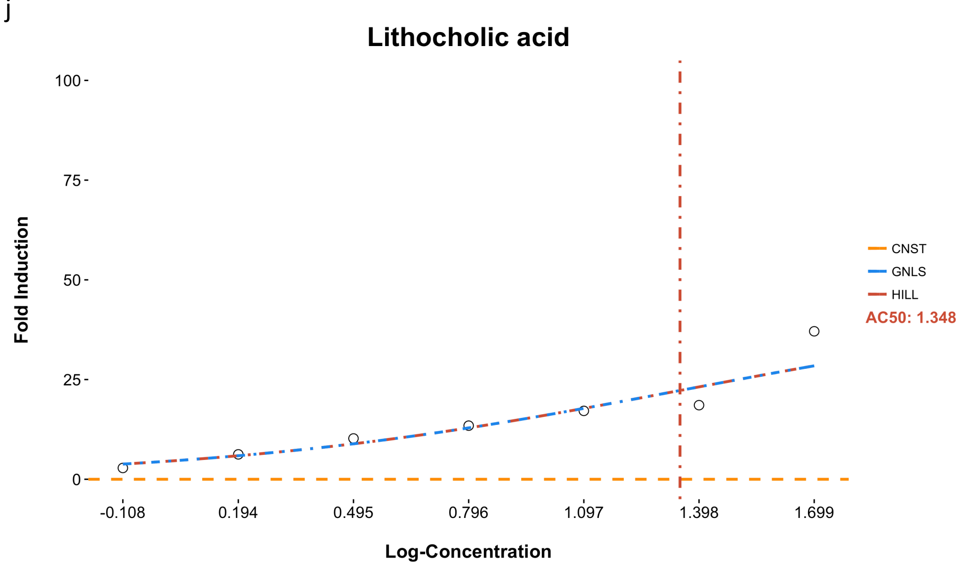

Lithocholic acid
Fold Induction
100
75
50
25
0
-0.108
0.194
0.495
0.796
1.097
1.398
1.699
Log-Concentration
CNST
GNLS
HILL
AC50: 1.348

k

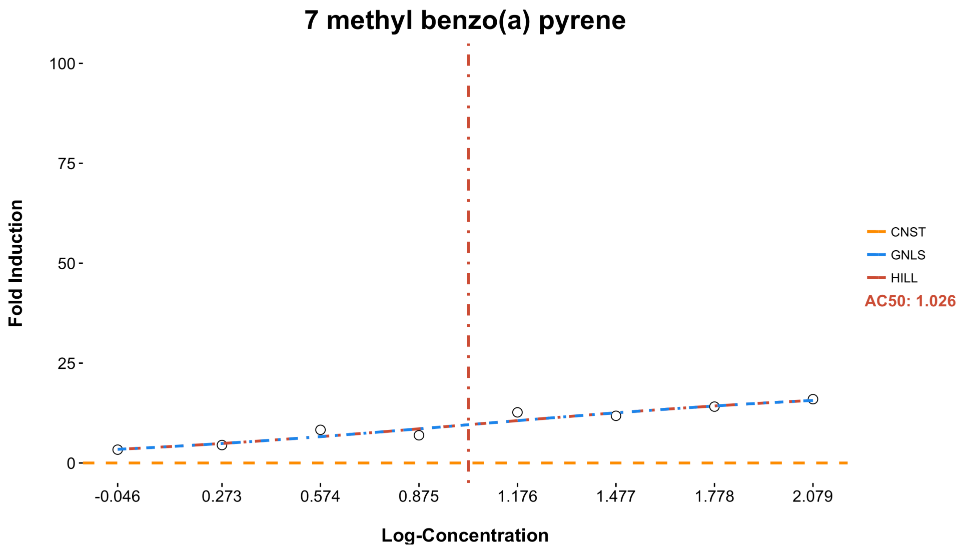
7 methyl benzo(a) pyrene
100
75
50
25
0
Fold Induction
-0.046
0.273
0.574
0.875
1.176
1.477
1.778
2.079
Log-Concentration
CNST
GNLS
HILL
AC50: 1.026

l

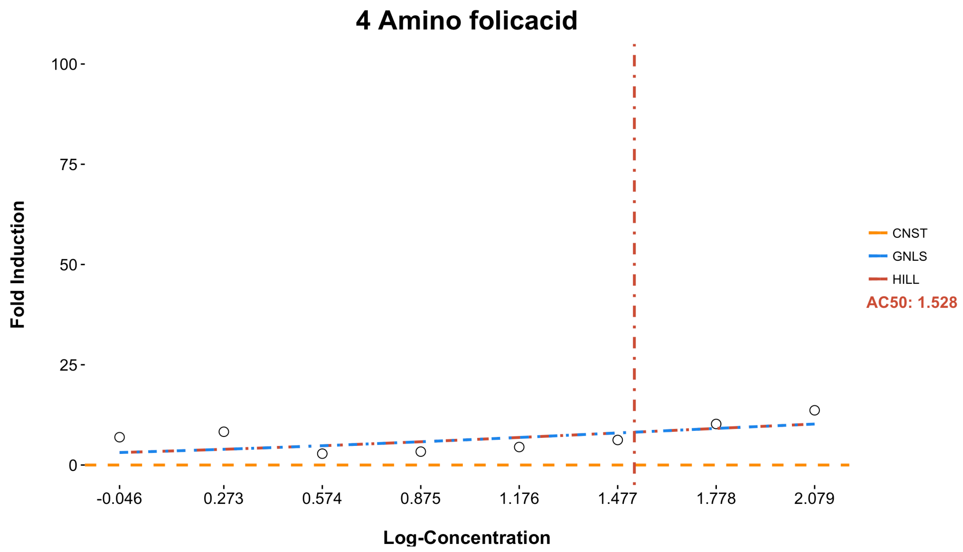
4 Amino folicacid
100
75
50
25
0
Fold Induction
-0.046
0.273
0.574
0.875
1.176
1.477
1.778
2.079
Log-Concentration
CNST
GNLS
HILL
AC50: 1.528

m

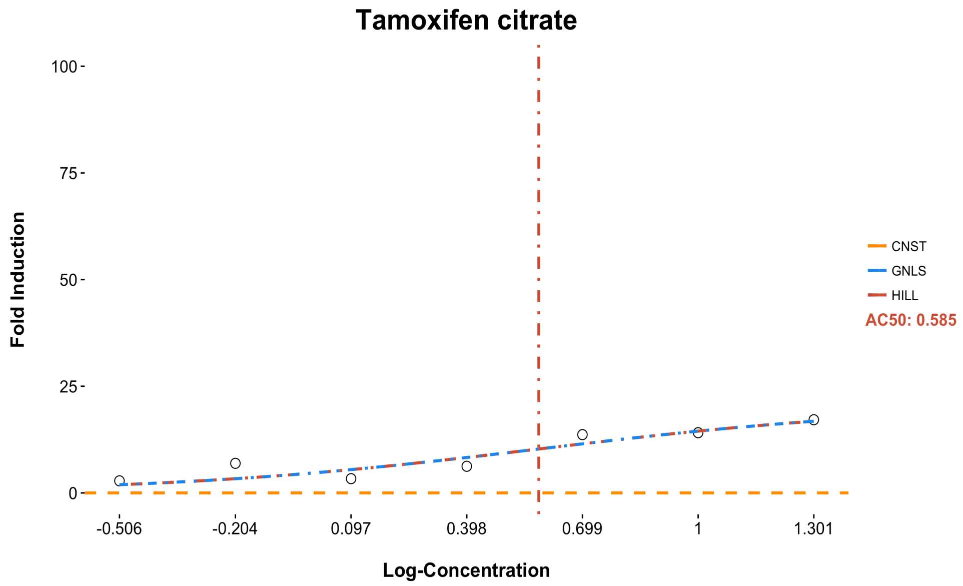
Tamoxifen citrate
100
75
50
25
0
Fold Induction
-0.506
-0.204
0.097
0.398
0.699
1
1.301
Log-Concentration
CNST
GNLS
HILL
AC50: 0.585

n

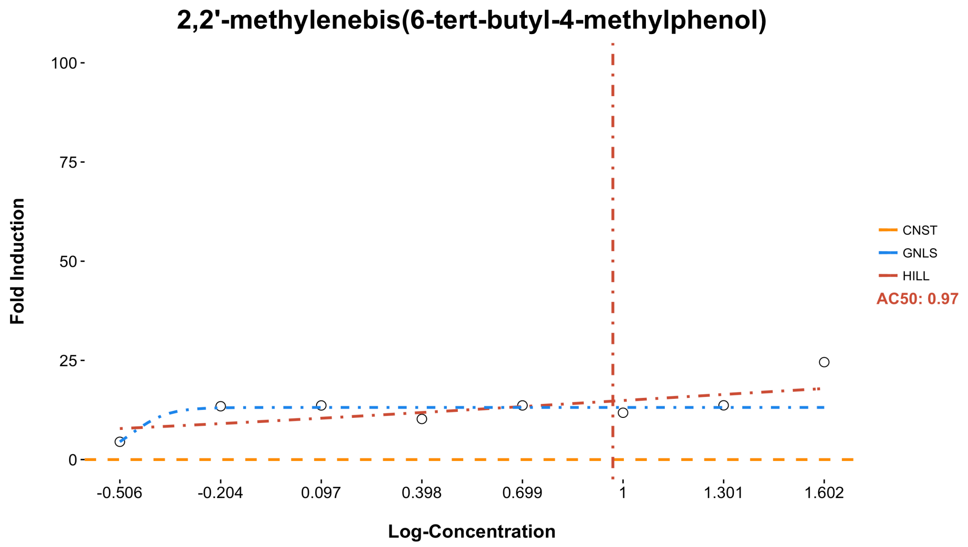
2,2'-methylenebis(6-tert-butyl-4-methylphenol)
100
75
50
25
0
Fold Induction
-0.506
-0.204
0.097
0.398
0.699
1
1.301
1.602
Log-Concentration
CNST
GNLS
HILL
AC50: 0.97

o

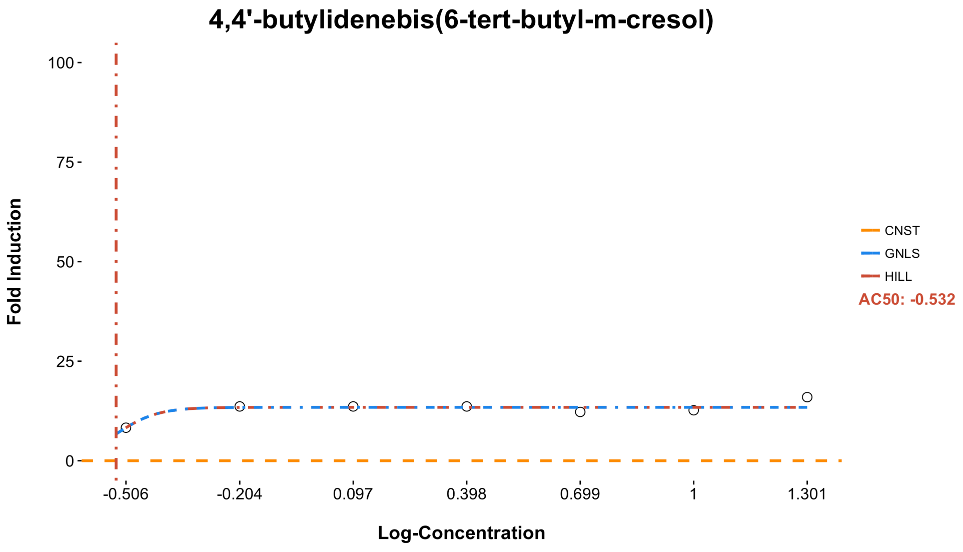
4,4'-butylidenebis(6-tert-butyl-m-cresol)
100
75
50
25
0
Fold Induction
-0.506
-0.204
0.097
0.398
0.699
1
1.301
Log-Concentration
CNST
GNLS
HILL
AC50: -0.532

p

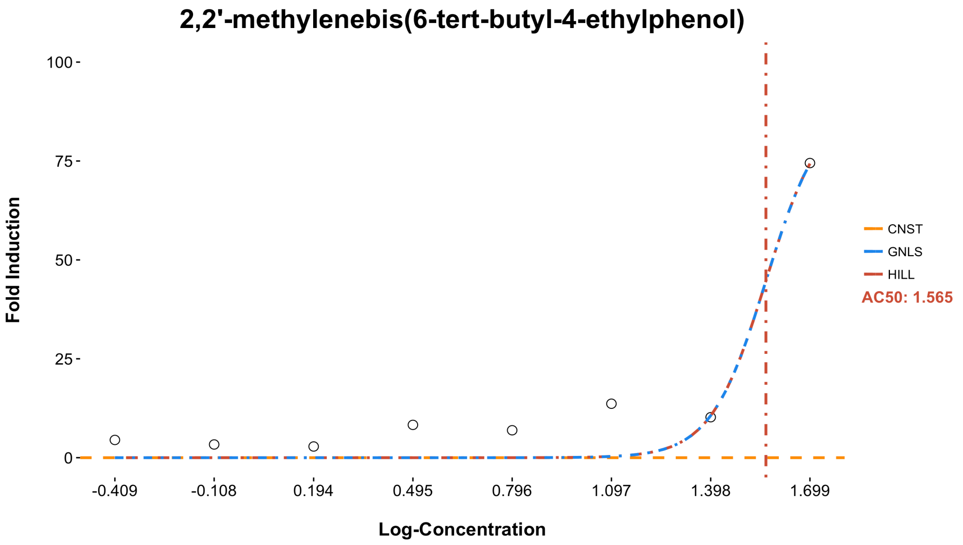
2,2'-methylenebis(6-tert-butyl-4-ethylphenol)
100
75
50
25
0
Fold Induction
-0.409
-0.108
0.194
0.495
0.796
1.097
1.398
1.699
Log-Concentration
CNST
GNLS
HILL
AC50: 1.565

q

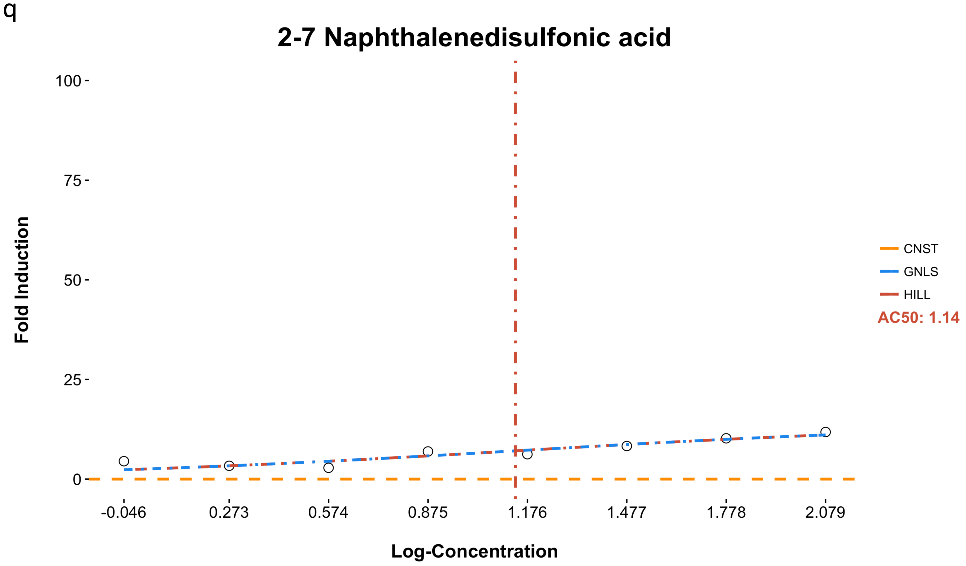

2-7 Naphthalenedisulfonic acid
Fold Induction
100
75
50
25
0
-0.046
0.273
0.574
0.875
1.176
1.477
1.778
2.079
Log-Concentration
CNST
GNLS
HILL
AC50: 1.14

r

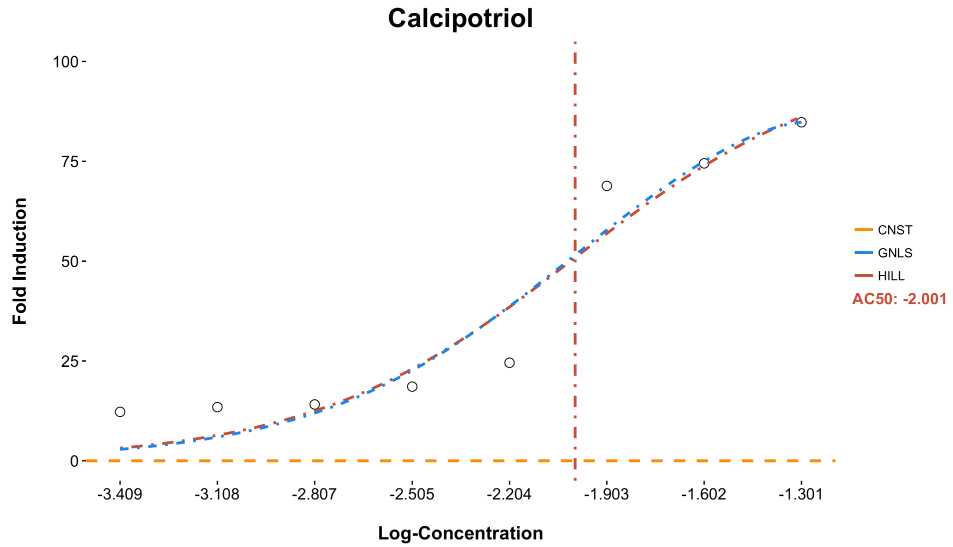

Calcipotriol
Fold Induction
100
75
50
25
0
-3.409
-3.108
-2.807
-2.505
-2.204
-1.903
-1.602
-1.301
Log-Concentration
CNST
GNLS
HILL
AC50: -2.001

s

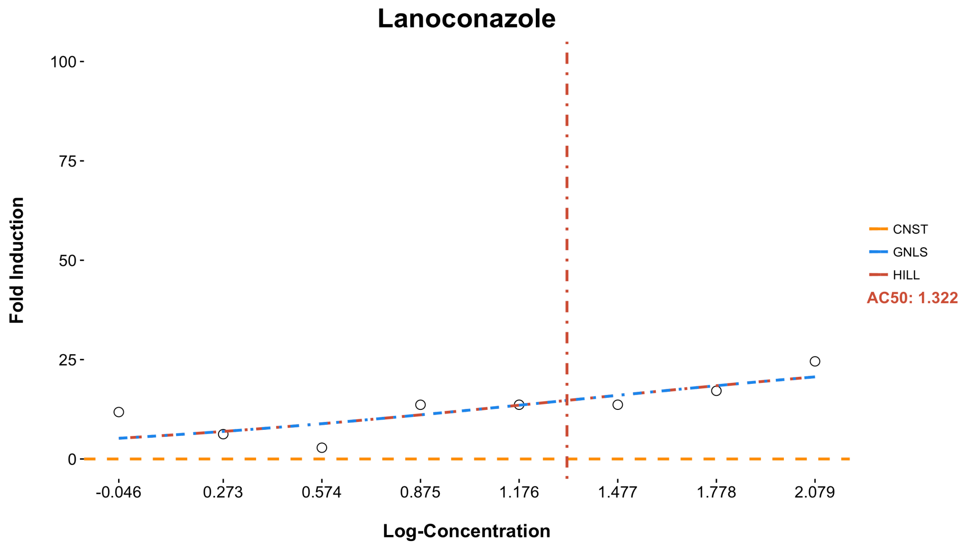
Lanoconazole
100
75
50
25
0
Fold Induction
-0.046
0.273
0.574
0.875
1.176
1.477
1.778
2.079
Log-Concentration
CNST
GNLS
HILL
AC50: 1.322

t

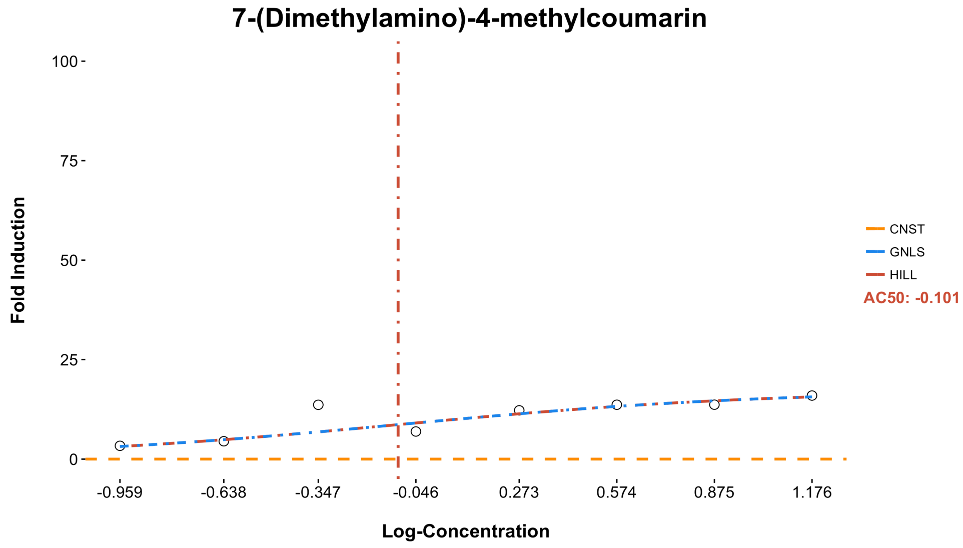
7-(Dimethylamino)-4-methylcoumarin
100
75
50
25
0
Fold Induction
-0.959
-0.638
-0.347
-0.046
0.273
0.574
0.875
1.176
Log-Concentration
CNST
GNLS
HILL
AC50: -0.101

u

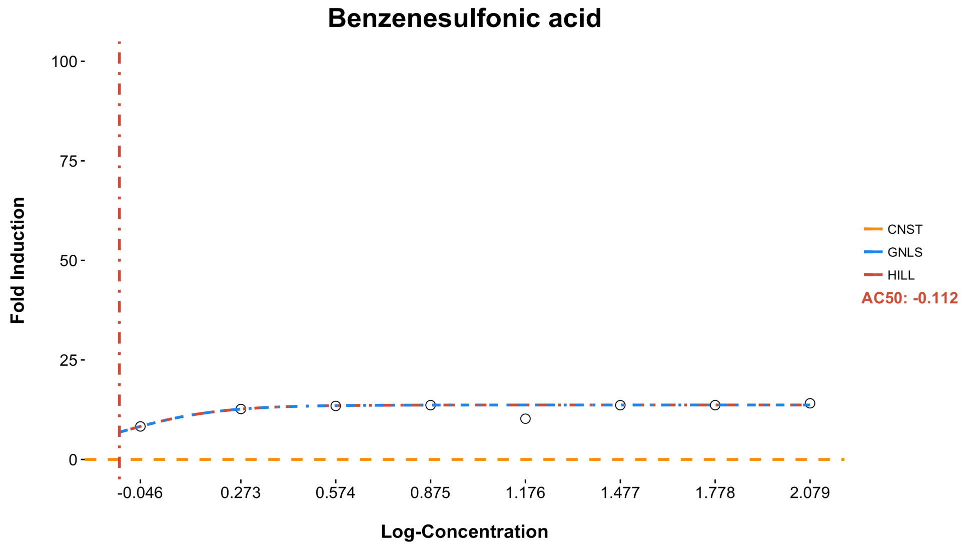
Benzenesulfonic acid
100
75
50
25
0
Fold Induction
-0.046
0.273
0.574
0.875
1.176
1.477
1.778
2.079
Log-Concentration
CNST
GNLS
HILL
AC50: -0.112

v

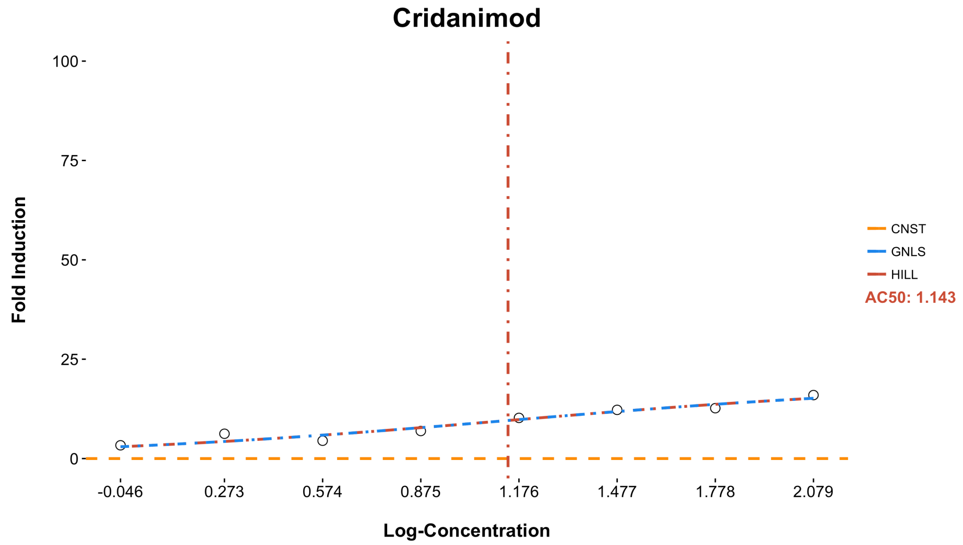
Cridanimod
100
75
50
25
0
Fold Induction
-0.046
0.273
0.574
0.875
1.176
1.477
1.778
2.079
Log-Concentration
CNST
GNLS
HILL
AC50: 1.143

Supplementary Figure S6. Shows transient transactivation dose response curves for all 21 agonists and vitamin D_3 (a-v). The normalization method used is quantile normalization, which uses rankings of the data and averages to adjust the data across distributions. IC50 values were derived from Hill and Gain-Loss models of dose-response curves generated using the TCPL R package

VDR Antagonists

a

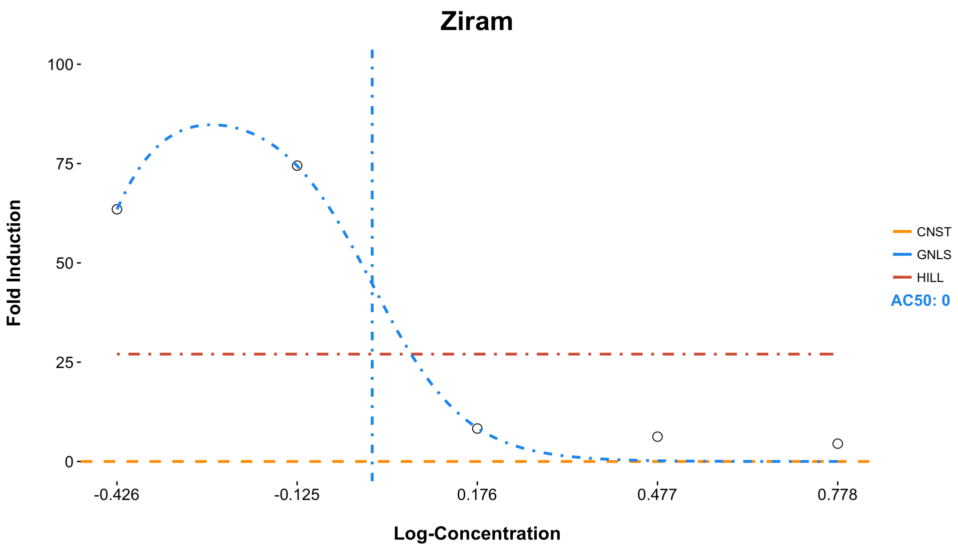

b

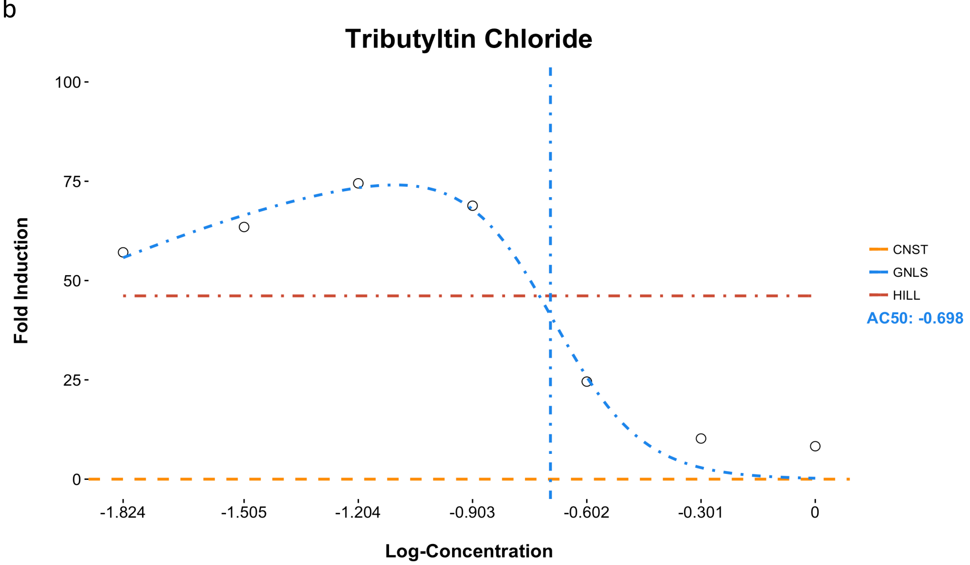

c

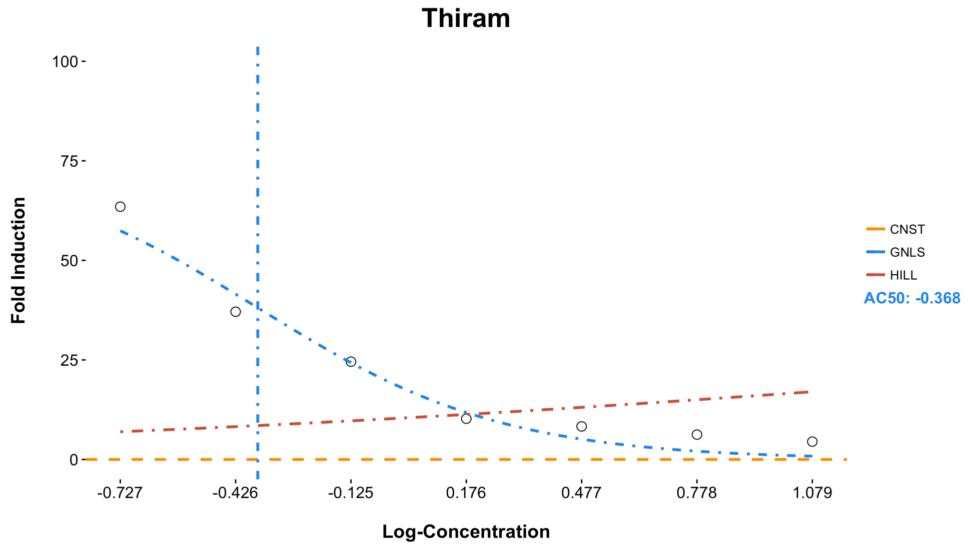
Thiram
100
75
50
25
0
Fold Induction
-0.727
-0.426
-0.125
0.176
0.477
0.778
1.079
Log-Concentration
CNST
GNLS
HILL
AC50: -0.368

d

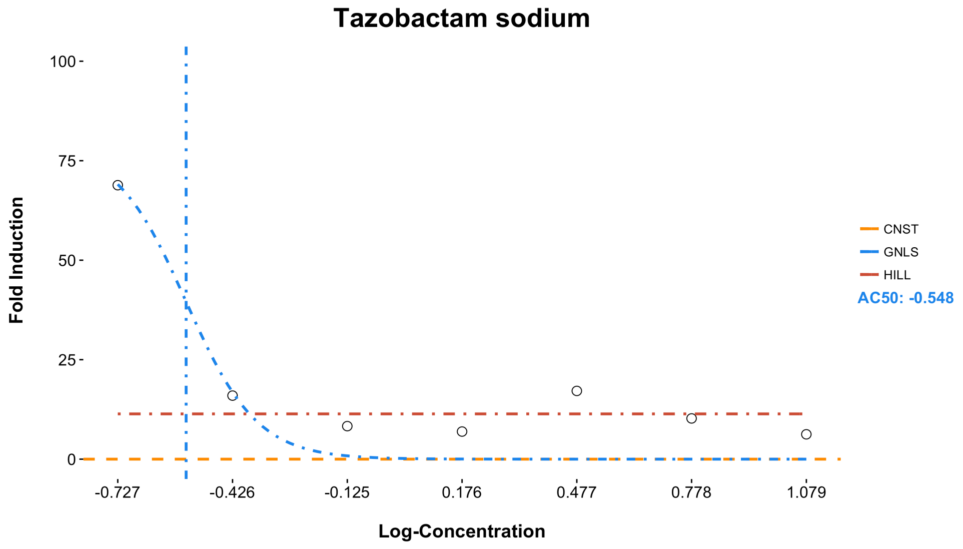
Tazobactam sodium
100
75
50
25
0
Fold Induction
-0.727
-0.426
-0.125
0.176
0.477
0.778
1.079
Log-Concentration
CNST
GNLS
HILL
AC50: -0.548

e

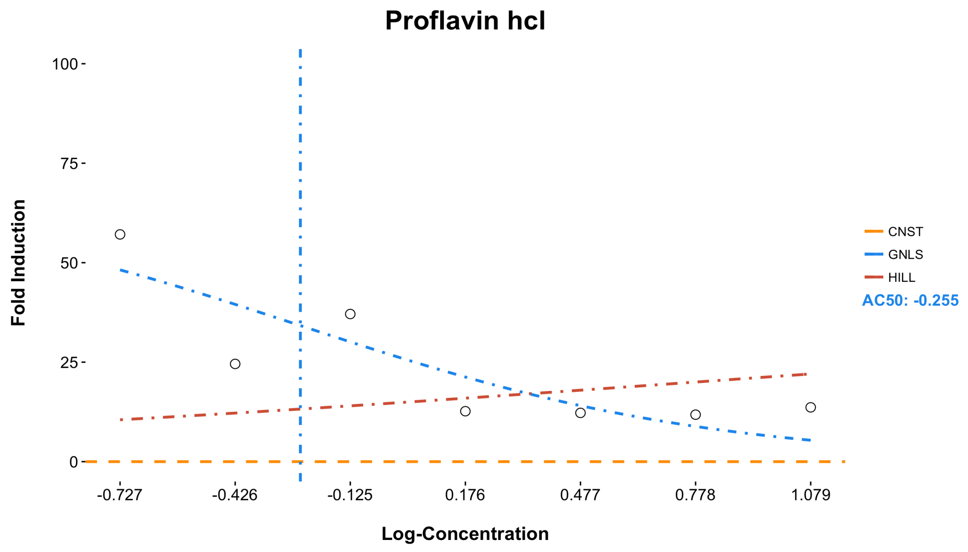

Proflavin hcl
100
75
50
25
0
Fold Induction
-0.727
-0.426
-0.125
0.176
0.477
0.778
1.079
Log-Concentration
CNST
GNLS
HILL
AC50: -0.255

f

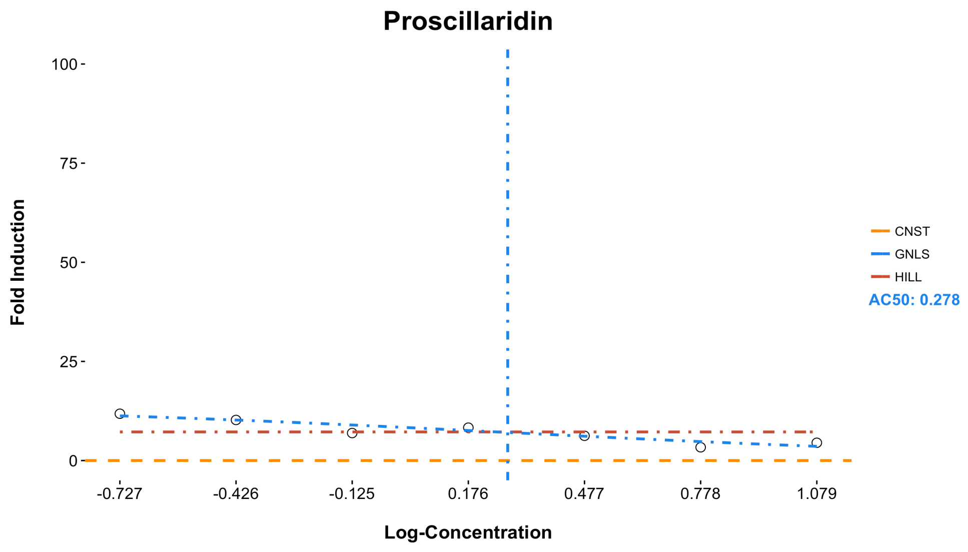

Proscillaridin
100
75
50
25
0
Fold Induction
-0.727
-0.426
-0.125
0.176
0.477
0.778
1.079
Log-Concentration
CNST
GNLS
HILL
AC50: 0.278

g

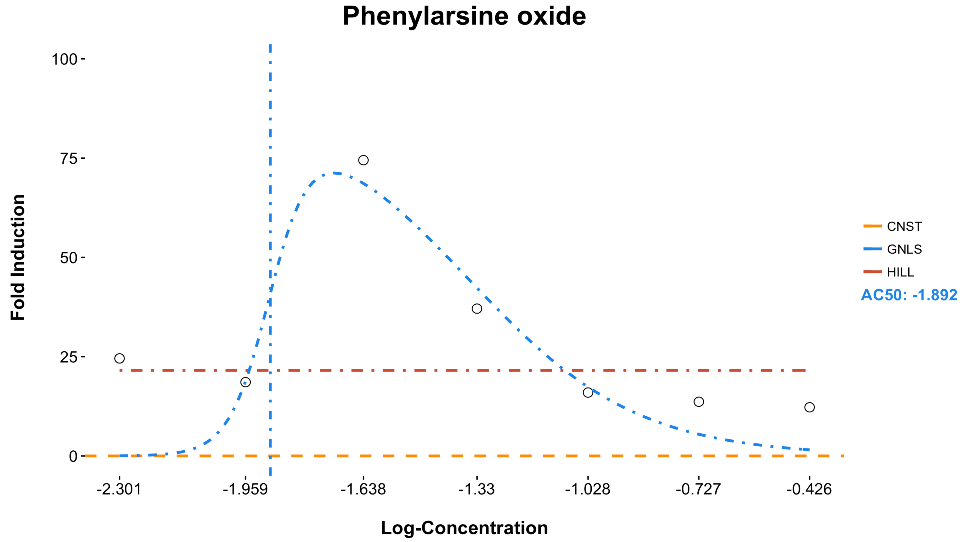

Phenylarsine oxide
Fold Induction
100
75
50
25
0
-2.301
-1.959
-1.638
-1.33
-1.028
-0.727
-0.426
Log-Concentration
CNST
GNLS
HILL
AC50: -1.892

h

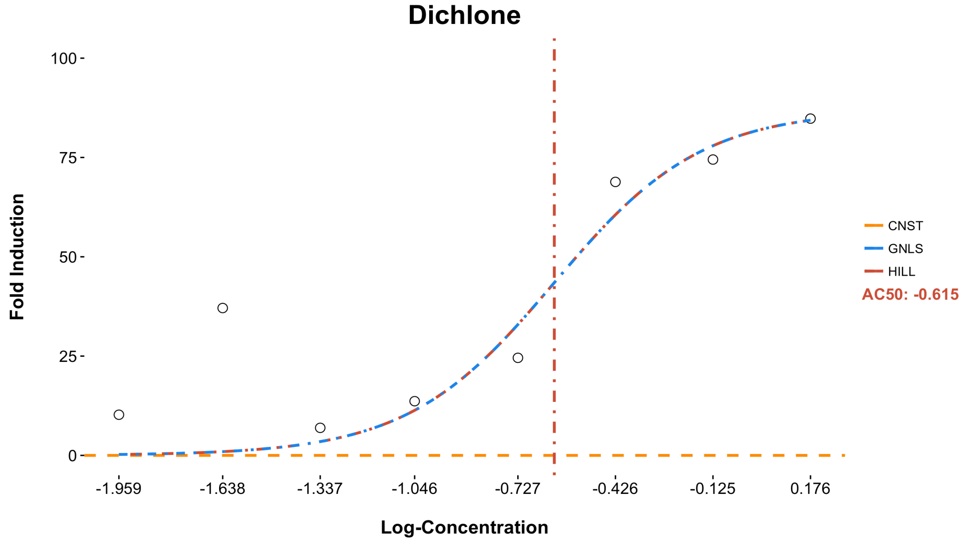

Dichlone
Fold Induction
100
75
50
25
0
-1.959
-1.638
-1.337
-1.046
-0.727
-0.426
-0.125
0.176
Log-Concentration
CNST
GNLS
HILL
AC50: -0.615

i

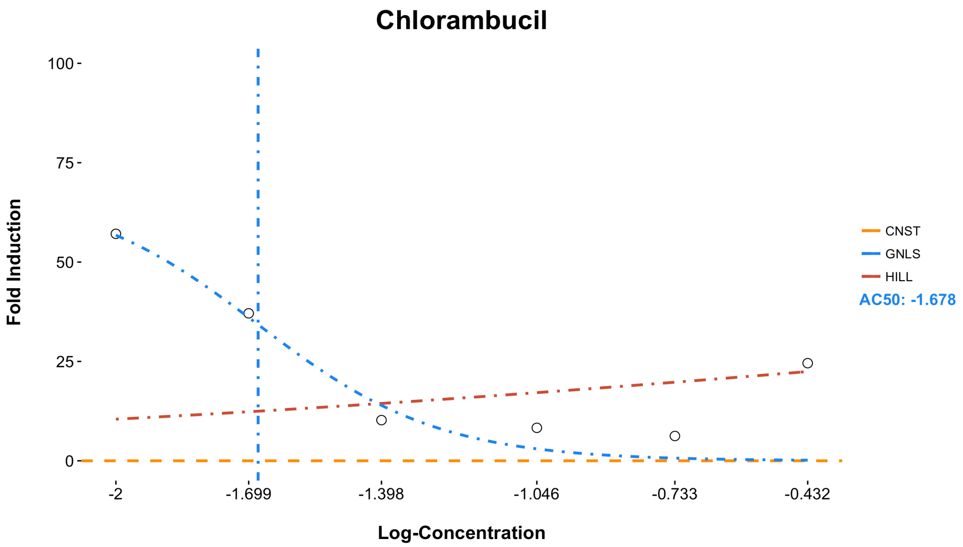
Chlorambucil
100
75
50
25
0
Fold Induction
-2
-1.699
-1.398
-1.046
-0.733
-0.432
Log-Concentration
CNST
GNLS
HILL
AC50: -1.678

j

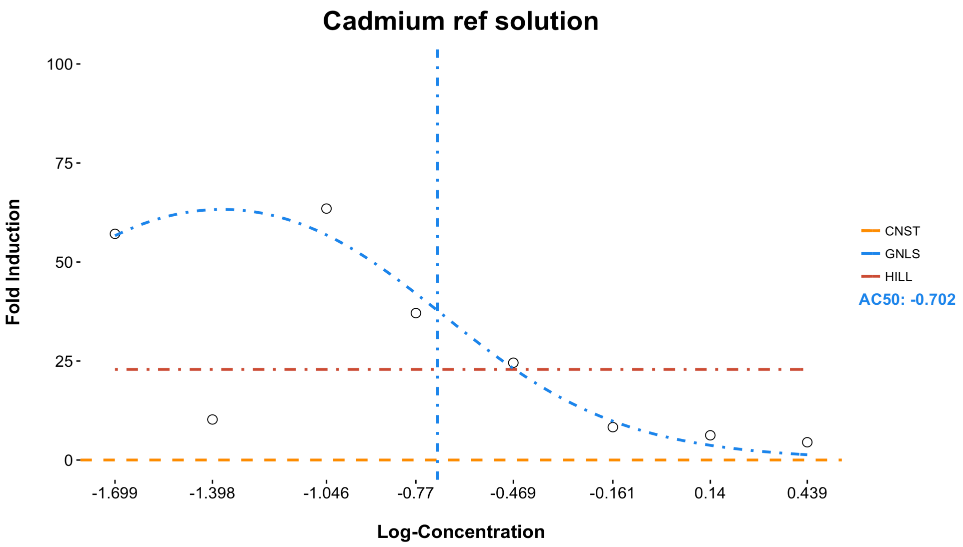
Cadmium ref solution
100
75
50
25
0
Fold Induction
-1.699
-1.398
-1.046
-0.77
-0.469
-0.161
0.14
0.439
Log-Concentration
CNST
GNLS
HILL
AC50: -0.702

k

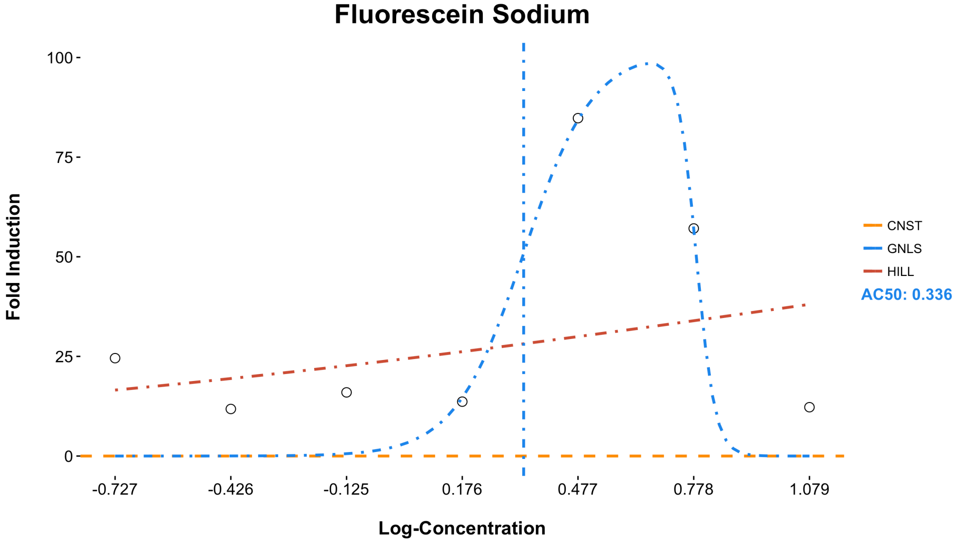
Fluorescein Sodium
100
75
50
25
0
Fold Induction
-0.727
-0.426
-0.125
0.176
0.477
0.778
1.079
Log-Concentration
CNST
GNLS
HILL
AC50: 0.336

l

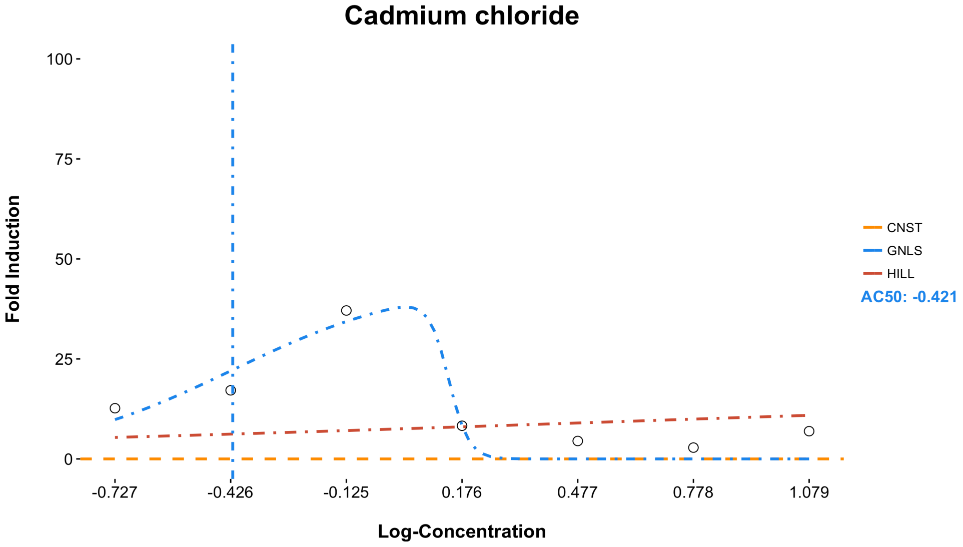
Cadmium chloride
100
75
50
25
0
Fold Induction
-0.727
-0.426
-0.125
0.176
0.477
0.778
1.079
Log-Concentration
CNST
GNLS
HILL
AC50: -0.421

m

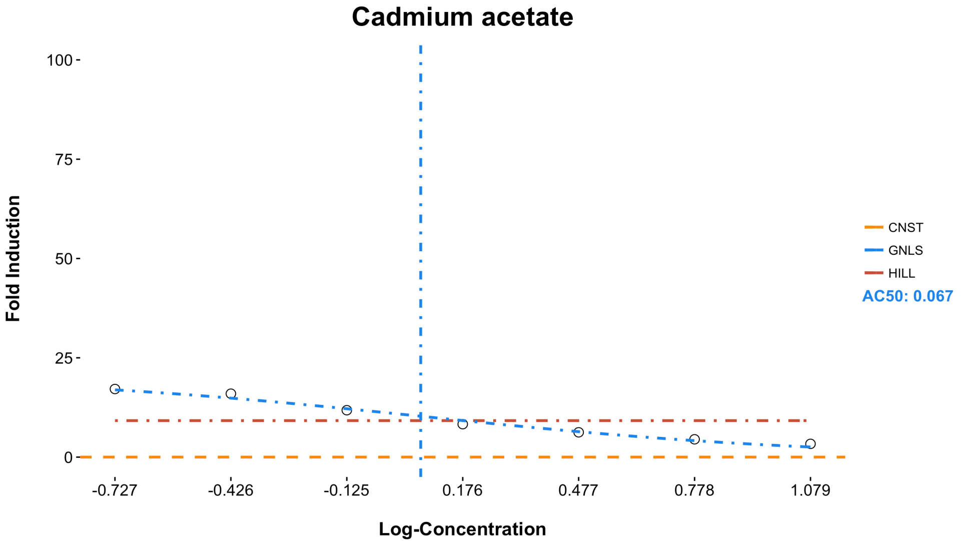
Cadmium acetate
Fold Induction
100
75
50
25
0
-0.727
-0.426
-0.125
0.176
0.477
0.778
1.079
Log-Concentration
CNST
GNLS
HILL
AC50: 0.067

n

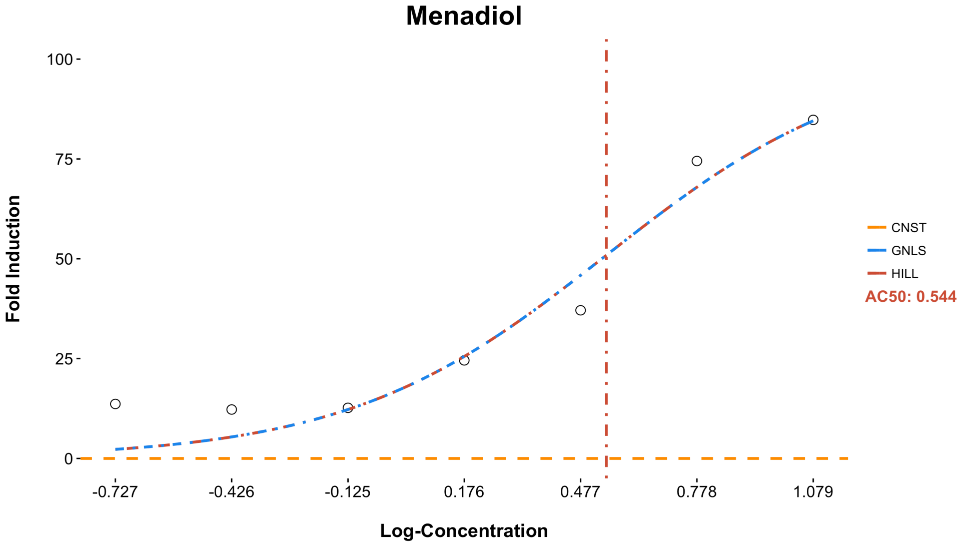
Menadiol
Fold Induction
100
75
50
25
0
-0.727
-0.426
-0.125
0.176
0.477
0.778
1.079
Log-Concentration
CNST
GNLS
HILL
AC50: 0.544

o

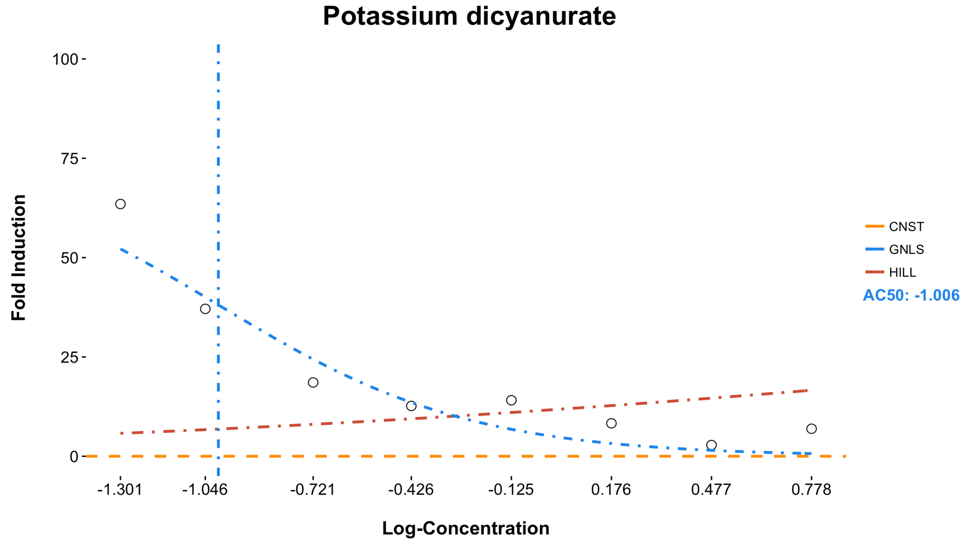

Potassium dicyanurate
Fold Induction
100
75
50
25
0
-1.301
-1.046
-0.721
-0.426
-0.125
0.176
0.477
0.778
Log-Concentration
CNST
GNLS
HILL
AC50: -1.006

p

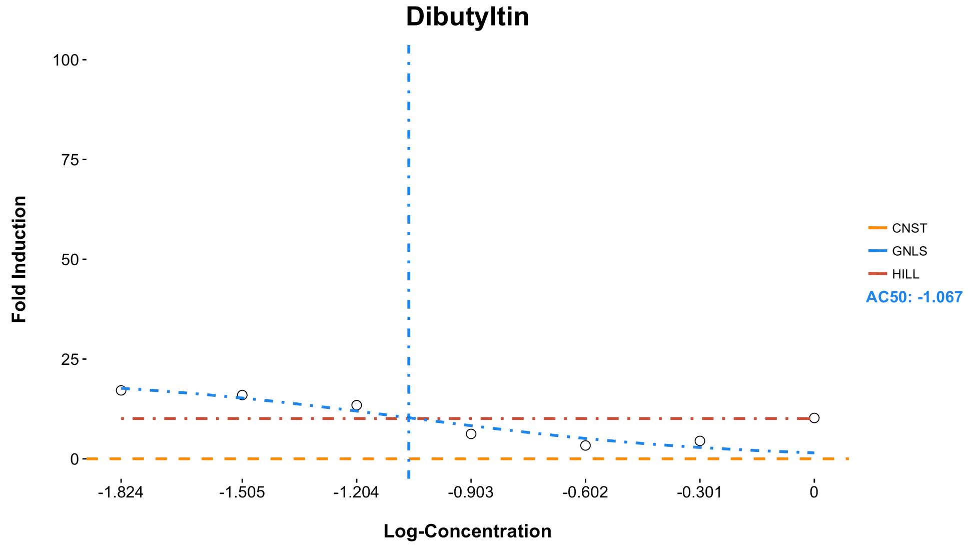

Dibutyltin
Fold Induction
100
75
50
25
0
-1.824
-1.505
-1.204
-0.903
-0.602
-0.301
0
Log-Concentration
CNST
GNLS
HILL
AC50: -1.067

q

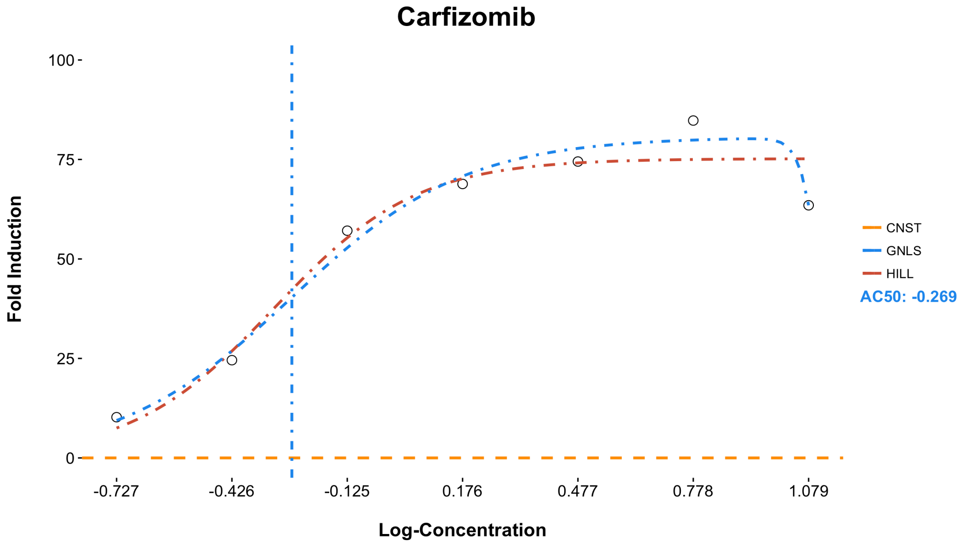
Carfizomib
100
75
50
25
0
Fold Induction
-0.727
-0.426
-0.125
0.176
0.477
0.778
1.079
Log-Concentration
CNST
GNLS
HILL
AC50: -0.269

r

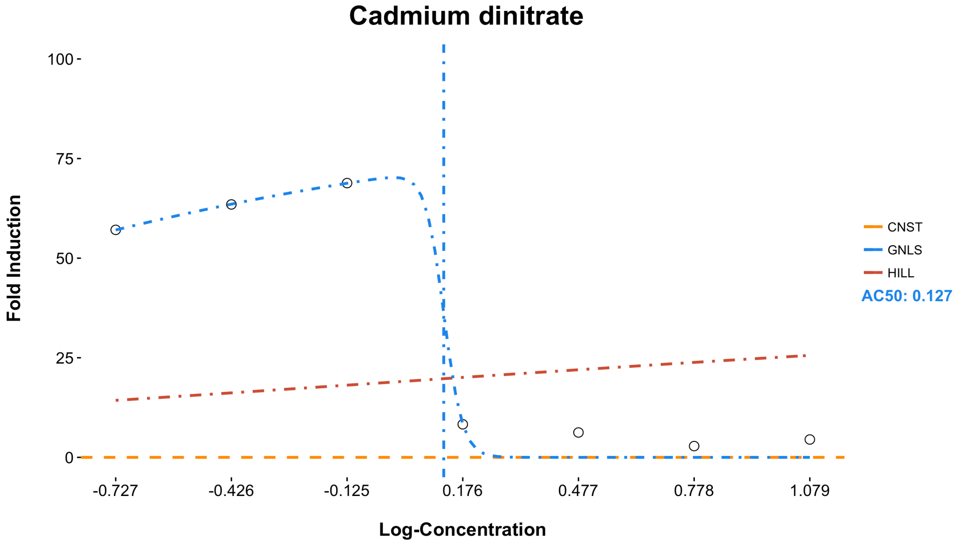
Cadmium dinitrate
100
75
50
25
0
Fold Induction
-0.727
-0.426
-0.125
0.176
0.477
0.778
1.079
Log-Concentration
CNST
GNLS
HILL
AC50: 0.127

s

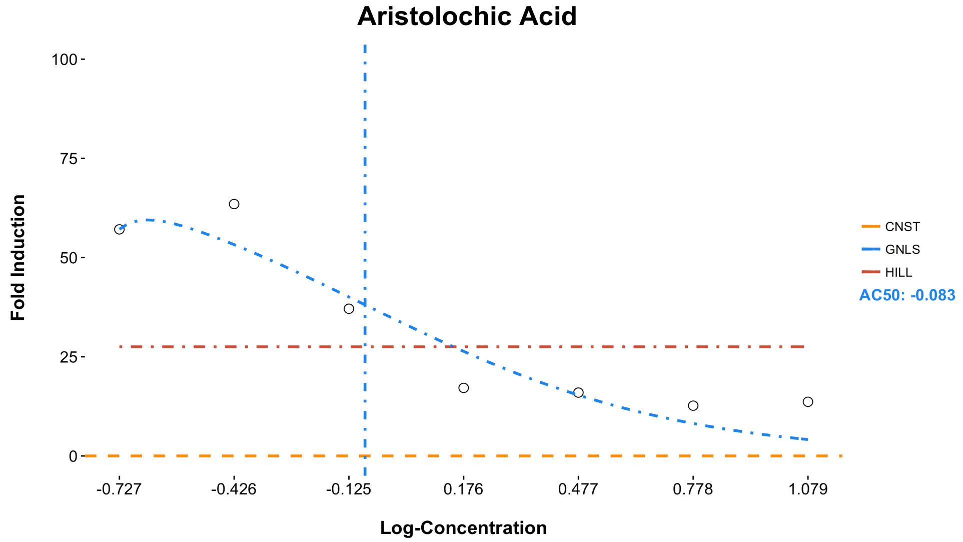

Supplementary Figure S7. Shows transient transactivation dose response curves for all 19 antagonists (a-s). The normalization method used is quantile normalization, which uses rankings of the data and averages to adjust the data across distributions. IC50 values were derived from Hill and Gain-Loss models of dose-response curves generated using the TCPL R package
